# Comparative Structural and Functional Analysis of Bunyavirus and Arenavirus Cap-Snatching Endonucleases

**DOI:** 10.1371/journal.ppat.1005636

**Published:** 2016-06-15

**Authors:** Juan Reguera, Piotr Gerlach, Maria Rosenthal, Stephanie Gaudon, Francesca Coscia, Stephan Günther, Stephen Cusack

**Affiliations:** 1 European Molecular Biology Laboratory, Grenoble Outstation, 71 Avenue des Martyrs, CS90181, 38042 Grenoble Cedex 9, France; 2 Unit of Virus-Host Cell Interactions (UMI 3265), Univ. Grenoble-Alpes-EMBL-CNRS, 71 Avenue des Martyrs, CS90181, 38042 Grenoble Cedex 9, France; 3 Department of Virology, Bernhard-Nocht-Institute for Tropical Medicine, Hamburg, Germany; Institut Pasteur, FRANCE

## Abstract

Segmented negative strand RNA viruses of the arena-, bunya- and orthomyxovirus families uniquely carry out viral mRNA transcription by the cap-snatching mechanism. This involves cleavage of host mRNAs close to their capped 5′ end by an endonuclease (EN) domain located in the N-terminal region of the viral polymerase. We present the structure of the cap-snatching EN of Hantaan virus, a bunyavirus belonging to hantavirus genus. Hantaan EN has an active site configuration, including a metal co-ordinating histidine, and nuclease activity similar to the previously reported La Crosse virus and Influenza virus ENs (orthobunyavirus and orthomyxovirus respectively), but is more active in cleaving a double stranded RNA substrate. In contrast, Lassa arenavirus EN has only acidic metal co-ordinating residues. We present three high resolution structures of Lassa virus EN with different bound ion configurations and show in comparative biophysical and biochemical experiments with Hantaan, La Crosse and influenza ENs that the isolated Lassa EN is essentially inactive. The results are discussed in the light of EN activation mechanisms revealed by recent structures of full-length influenza virus polymerase.

## Author Summary

Segmented negative strand viruses (sNSV) such as Influenza, Lassa or Hantaan viruses are responsible for a large number of severe human infectious diseases. Currently, there are vaccines and antiviral treatments available for influenza but none for the infections caused by other sNSV. All carry out transcription by the cap-snatching mechanism, which requires the action of a metal ion dependent endonuclease (EN), a domain within their large viral polymerases. Here we provide the crystal structure of the Hantaan virus (family *Bunyaviridae*) and Lassa virus (family *Arenaviridae*) cap-snatching ENs in complex with manganese and a comparative functional study of their catalytic activity. Despite the high structural homology between the two ENs a few changes in the active site, involving a catalytic histidine, cause a different binding of the metal ions with dramatic consequences for their in vitro activity. Hantaan EN binds the metal ions as Influenza A (family *Orthomyxoviridae*) and LACV (family *Bunyaviridae*) ENs and all three are active in vitro. In contrast Lassa virus EN is inactive in the same experimental conditions. We can now classify sNSV into two functionally distinct groups (His+ and His- ENs), providing a broad view of the sNSV cap-snatching ENs properties that will be determinant for the comprehensive development of broad-spectrum antiviral drugs. These results also have implications for the viral transcription regulation in the light of recent studies on full-length sNSV polymerases.

## Introduction

Segmented negative strand viruses (sNSVs) represent one of the most threatening groups of emerging viruses for global health [1]. They are classified in three main families: *Orthomyxoviridae*, *Bunyaviridae* and *Arenaviridae* with respectively six to eight, three and two genome segments [2]. Seasonal and pandemic influenza A virus (IAV, orthomyxovirus) strains rapidly propagate worldwide with human to human transmission being the key factor for spread. In contrast, arenaviruses (e.g. Lassa virus) or bunyaviruses (e.g. Hantaan, La Crosse, Rift Valley, Crimean Congo Haemorrhagic viruses), as well as some highly pathogenic avian influenza strains, are zoonotic viruses that result in generally limited outbreaks through contact with animal vectors but with high mortality rates and lack of effective treatments. The future spread of some of these infectious agents from their traditional geographical niches due to vector species redistribution arising through climate change is a potential threat [3,4], emphasising the need to develop new, ideally broad-spectrum, drugs against sNSV zoonotic viral diseases.

Despite the diversity in the infectious cycles of sNSVs there are common mechanisms that can be potentially targeted for broad spectrum inhibitors, such as genome and mRNA synthesis by the conserved RNA dependent RNA polymerase (RdRpol) or their characteristic cap-snatching transcription mechanism [5–8]. This mechanism, most extensively characterized for IAV virus, involves the recognition of capped cellular mRNAs by a cap-binding domain located in the polymerase and its subsequent cleavage 10–14 nucleotides downstream by the polymerase’s endonuclease (EN) to provide a primer for viral mRNA transcription [5,9]. The cap-binding and the EN domains were first identified in the IAV hetero-trimeric polymerase and are located in the middle region of the PB2 and the N-terminal region of the PA subunits respectively [10,11]. The recent crystal structures of influenza A and B heterotrimeric polymerases show the relative disposition of these two domains within the full RdRpol domains allowing an integrated structural model for the cap-snatching mechanism to be proposed for orthomyxoviruses [9,12]. Studies on La Crosse (LACV) bunyavirus and Lymphocytic Choriomeningitis (LCMV) arenavirus allowed the structural and functional characterization of the cap-snatching EN domains in the amino terminal region of their monomeric polymerases (L proteins) [13,14] and showed them to be essential for viral transcription. Similar results were subsequently obtained for Lassa arenavirus and the bunyaviruses Rift Valley Fever Virus (RVFV) and Crimean Congo Haemorragic Fever Virus (CCHFV) [15–18]. However the location of the putative cap-binding domain still remains elusive for bunya- and arenaviruses.

The sNSV cap-snatching ENs belong to the PD-D/ExK superfamily of cation dependent nucleases. The available structures of the influenza orthomyxovirus and LACV orthobunyavirus show the canonical conformation of the active site with two divalent metal ions directly coordinated by the acidic conserved residues of the PD and the D/ExK motifs as well as with a conserved histidine (His+ ENs). The two metal ions bind aligned towards the catalytic lysine [14]. The arenavirus EN crystal structures reported to date (LCMV and Lassa) are structurally homologous to LACV EN [13,16], but there are important differences in their active sites. The main divergence is that the metal co-ordinating histidine, conserved in most bunya- and orthomyxoviruses, is replaced by an acidic residue in arenavirus ENs (His- ENs). No metal ions were present in the LCMV EN structure [13] and the Lassa EN structure was reported with two magnesium ions in the active site coordinated by some catalytic residues through bridging water molecules, instead of the direct coordination shown by His+ ENs. The reported ion preference for the catalytic activity also changes, Lassa EN preferring magnesium and LCMV EN or His+ ENs preferring manganese [16].

Here we focus on two sNSV, Hantaan bunyavirus and Lassa arenavirus, that are both transmitted to humans by rodents and can cause severe haemorrhagic fevers with up to 50% fatality rates [19,20]. To demonstrate the presence of a cap-snatching endonuclease domain in hantavirus L proteins we determine the crystal structure of the isolated Hantaan virus EN in complex with Mn^2+^ ions and characterize its endonuclease activity. By comparing the activity and ion binding with the IAV (*Orthomyxoviridae*), LACV (*Orthobunyaviridae*) and Lassa (*Arenaviridae*) ENs we find that the catalytic histidine present in Hantaan and other His+ ENs correlates with high endonuclease activity. Subsequent structural characterization of the Lassa endonuclease with bound ions reveals an active site with a non-canonical coordination of the catalytic metal ions and this correlates with low intrinsic activity. Therefore the histidine of His+ ENs appears to promote the canonical binding of metal ions in the active site and is a determinant for efficient *in vitro* catalytic activity. These results are relevant for understanding possible differences in the mechanism of regulation of EN activity and have strong implications in the development of new antiviral drugs targeting transcription of sNSV.

## Results

### Crystal structure of Hantaan virus endonuclease in complex with Mn^2+^ ions

By sequence alignment the Hantaan virus EN is predicted to be at the N-terminus of the L protein [14]. We could express and purify protein constructs comprising residues 1–179 and 1–182 that crystallized with 2 mM MnCl_2_ as thick plates diffracting to 1.7 Å resolution. The structure was solved by SAD experiment (see Materials and Methods and Table 1). Each protein binds two Mn^2+^ ions in the active site in a similar fashion to that observed for LACV and influenza [10,14]. A third Mn^2+^ stabilises the interface between crystallographic symmetry related neighbour proteins (S1A Fig).

**Table 1:** Data refinement and collection statistics. In parenthesis are indicated the values for the last shell. The additives used in the sample and cryoprotectant solution are also indicated.

	Hantaan SAD	Hantaan natPDB: 5IZE	Lassa X1PDB: 5IZH	Lassa X2PDB: 5J1N	Lassa X3PDB: 5J1P
**Space group**	*P2*_*1*_	*P2*_*1*_	*P2*_*1*_ *2*_*1*_ *2*_*1*_	*P4*_*1*_ *2*_*1*_ *2*	*P4*_*1*_ *2*_*1*_ *2*
**Cell dimensions (Å)**	a = 41.0 b = 77.1	a = 41.2 b = 76.1	a = 58.75	a = b = 51.5	a = b = 51.42
	c = 63.5	c = 63.72	b = 60.13	c = 143.0	c = 144.18
	α = γ = 90	α = γ = 90	c = 132.78	α = β = γ = 90	α = β = γ = 90
	β = 102.9	β = 103.9	α = β = γ = 90		
**Resolution range (last shell) (Å)**	50–2.20 (2.26–2.2)	48–1.7 (1.75–1.7)	66.4–1.85 (1.89–1.85)	48.45–1.09 (1.12–1.09)	48.4–2.36 (2.46–2.36)
**Beamline**	ESRF ID23-1	ESRF id23-1	ESRF id14-4	ESRF id23-2	SOLEIL PROXIMA1
**Wavelength (Å)**	1.77120	0.984	0.939	0.976	0.976
**Completeness (last shell) (%)**	99.9 (100)	97.8 (94.0)	99.5 (99.9)	99.19 (96.7)	99.8 (100)
**Detector**	Pilatus	Pilatus	Pilatus	Pilatus	Pilatus
**R-meas (last shell)**	36.2 (117.5)	6.7 (103.0)	5.6 (72.6)	6.1 (70.5)	6.4 (120.8)
**I/σI (last shell)**	9.65 (2.28)	11.99 (1.34)	15.11 (1.84)	16.93 (1.73)	22.0 (1.56)
**Total reflexions**	375208	124483	193759	542244	57512
**Redundancy**	9.73 (9.73)	3.02 (3.01)	4.78 (4.74)	6.72 (2.9)	6.7 (5.86)
**SigAno (3.11–2.97 Å)**	0.969 (1)				
**No. of reflexions used in refinement (free reflections)**		39163 (1991)	38459 (2017)	76549 (4043)	8093 (411)
**R-factor (last shell)**		0.166 (0.36)	0.205 (0.329)	0.161 (0.272)	0.20374 (0.300)
**R-free (last shell)**		0.216 (0.43)	0.259 (0.373)	0.179 (0.272)	0.28861 (0.361)
**Total atoms in the structure**		3094	2932	1646	1379
**Protein atoms**		2809	2734	1416	1363
**Ligand atoms**		6	0	17	3
**Water molecules**		279	198	213	13
**Average B-value (Å** ^**2**^ **)**		31.0	42	13	65
**Ramachandran plot favored regions**		99%	98%	99%	99%
**Ramachandran plot allowed regions**		1%	2%	1%	1%
**Bond distance deviations from ideal (Å)**		0.009	0.012	0.010	0.009
**Angles deviations from ideal(** ^**o**^ **)**		1.365	1.478	1.498	1.470
**Sample solution**	2mM MnCl_2_	2mM MnCl_2_	2mM MnCl_2_	2mM MnCl_2_	2mM MnCl_2_
				10mM GTP	5mM DPBA
**Cryoprotectant solution**	2mM MnCl_2_	2mM MnCl_2_	5mM MnCl_2_	5mM MnCl_2_	5mM MnCl_2_
				10mM GTP	5mM DPBA

**Fig 1:**
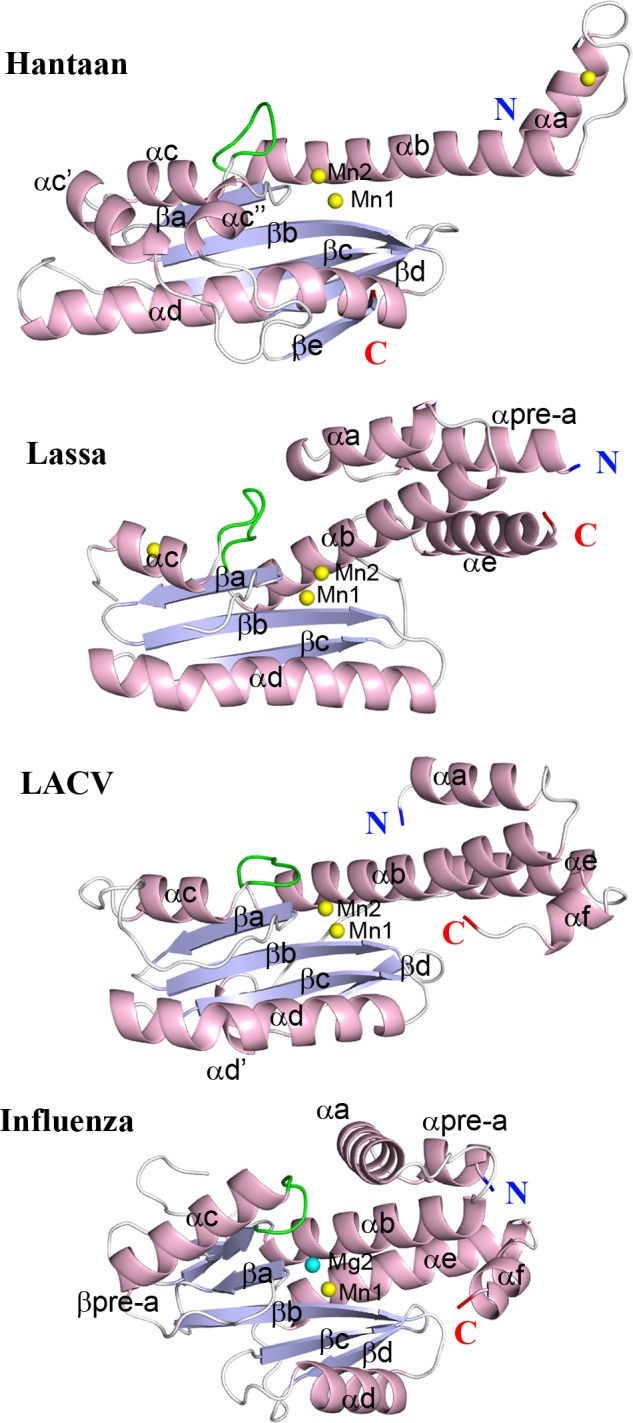
Crystal structure of Hantaan and Lassa ENs in comparison with LACV and Influenza. Cartoon representation of the crystal structures of Hantaan, Lassa (form X3), LACV (PDB: 2xi5) and IAV (PDB: 4avq) cap-snatching ENs. The alpha helices are coloured in light pink, the beta strands in light blue and the loops in light grey. The conserved flexible loop involved in the active site is highlighted in green. The same nomenclature is used to label homologous secondary structures. The metal ions are shown as yellow (Mn^2+^) or cyan (Mg^2+^) small spheres. The catalytic metal ions are labelled (Mn1-Mn2/Mg2) indicating the active site position. Note that the orientation of the catalytic ions changes between arenavirus Lassa EN and the rest of ENs.

**Fig 2:**
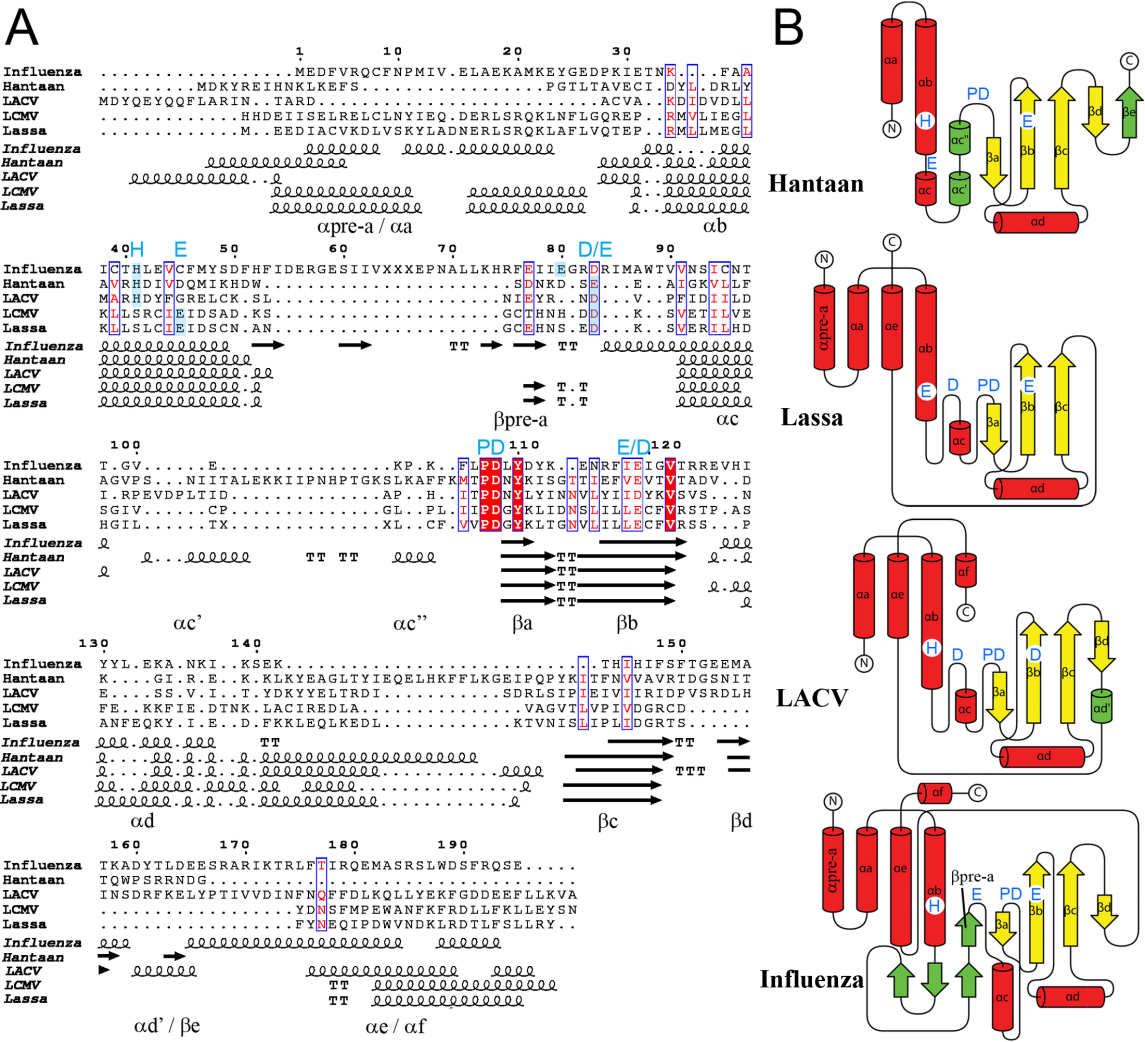
Structural alignments of sNSV cap-snatching ENs. **A,** The amino acid sequences and secondary structures of each EN aligned using PDBeFold [23] and represented using the ESPript server. Secondary structures are labelled like in Fig 1. The structures aligned are IAV (PDB: 4avq), Hantaan (nat), LACV (PDB: 2xi5), LCMV (PDB: 3jsb) and Lassa ENs (form X3). The ion coordinating catalytic residues are highlighted by blue labels on the top of the alignment. The histidine conserved in orthomyxo- and bunyavirus and the glutamate conserved in arenavirus are highlighted by blue shadows. The Influenza E80 and functionally homologous residues on the flexible loops are also highlighted by blue shadows and labelled (D/E). **B,** Topological diagram of the various ENs with the common core alpha helices as red cylinders and the beta sheets as yellow arrows. The secondary structures are labelled as in panel A. The specific insertions for each EN are coloured in green. The active site metal ion binding residues are indicated as in panel A.

The structure of the Hantaan virus cap-snatching EN (molecule A) is shown in comparison with Lassa (form X3, see below), LACV orthobunyavirus (PDB: 2xi7) and IAV H1N1 virus (PDB: 4avq) in Fig 1. Despite sequence identities below 20%, all structures present root mean square deviations of around 3.5 Å for at least 98 residues alignment (Dalilite) [21] (S1 Table). Overall, Hantaan EN conserves the two lobe shape of LACV but with the active site, which lies between the two lobes, being more accessible (S2 Fig). The central β-sheet made by the four conserved strands βa-d is extended by βe until the C-terminus of the construct (Figs 2A, 2B and S1B). LACV EN does not have an equivalent of this βe strand and instead, the 30 C-terminal residues of LACV fold into three α-helices, two integrated into the helical bundle which also includes the N-terminal α-helices (S1B Fig). The central β-sheet is surrounded by the conserved helices αb-e. A specific insertion between helix αc and the β-sheet, consisting of helices αc’ and αc” partially covers the β-sheet on the catalytic side (Figs 1 and 2). The flexible loop linking αb and αc (highlighted in green in Fig 1), harbouring a catalytic acidic residue (see below), plays the same structural and functional role as in IAV and LACV ENs. In Hantaan EN helix αa points outwards compared to the other ENs (Fig 1). This conformation, identical for the two molecules in the asymmetric unit, is stabilized by crystallographic contacts partially mediated by a shared, non-active site Mn^2+^ ion (S1A Fig), and might also be a consequence of the lack of the C-terminal α-helices stabilising the helical bundle, thus allowing helix αa to move (S1B Fig).

A structure based alignment of sNSV ENs of known crystal structure illustrates not only the overall conservation of secondary structures and catalytic residues but also the specific features of each family (Fig 2A and 2B). The structures show an identical secondary structure organization in the central region, starting from helix αb and ending at strand βc. However the different lengths of helices αc and αd change the overall shape. The longer helix αc and much shorter helix αd of IAV EN confer a globular shape in contrast with the elongated shape of bunya- and arenavirus ENs (Figs 1 and 2). Hantaan EN has a unique two α-helix insertion after αc, whereas IAV has a longer unique insertion between helices αb and αc. The structural alignment of the N- and C-terminal regions is poor because of the different arrangement of terminal alpha helices building the helical bundle around conserved helix αb. The 182 residue long Hantaan construct has only 16 residues after strand βc instead of 45 in IAV or 50 in LACV. Thus our structure lacks part of the helical lobe (see [22], co-submission). However we were not able to express longer constructs with the wild-type sequence in *E*.*coli*.

**Fig 3:**
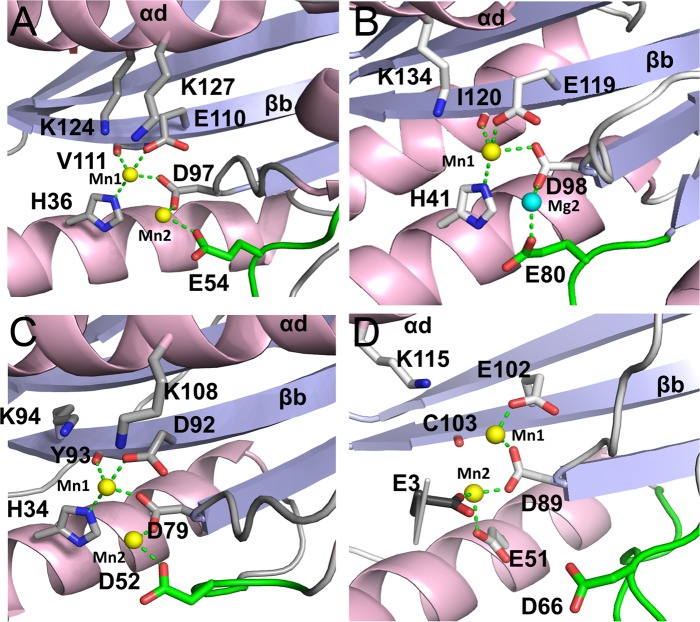
Structure of Hantaan and Lassa ENs in comparison with LACV and Influenza active sites and ion coordination. Structure of the active sites from ENs showed in Fig 1. The conserved catalytic residues are shown as sticks and the metal ions as yellow (Mn^2+^) or cyan (Mg^2+^) spheres and their coordination by dashed green lines. In all cases the helix αd and the strand βb are labelled. **A,** Hantaan EN active site. **B,** IAV EN active site. In this structure (PDB: 4avq) a Mg^2+^ ion substitute the Mn2 found in other published Influenza endonuclease structures. The H41 side chain rotamer was corrected from the original PDB to properly coordinate Mn1 with NE2. **C,** LACV EN active site. **D,** Lassa EN active site (form X3). The residue E3, coordinating Mn2 from a symmetry related molecule, is shown in black sticks.

The active site of Hantaan EN structure is configured very similarly to that of LACV and IAV ENs with two divalent cations bound in a canonical way (Fig 3A, 3B and 3C). The two ions (denoted Mn1 and Mn2) were identified as Mn^2+^ by the anomalous scattering signal detected at their respective positions (S1C Fig). Mn1 is octahedrally coordinated by the side chains of amino acid residues H36, D97 (from the conserved PD motif) and E110, and the main chain carboxyl oxygen of V111. The putative catalytic lysine K124 (see below) is deployed from helix αd as is K134 in IAV (Fig 3A and 3B) whereas the LACV catalytic lysine (K94) is deployed from strand βb (Fig 3C). Mn2 is coordinated by D97 and E54 coming from the conserved flexible loop preceding helix αc. Together all these residues constitute the conserved bunya/orthomyxovirus motif H.PD.D/E.K. The octahedral coordination of each Mn^2+^ is completed by two and four water molecules for Mn1 and Mn2 respectively, one central water being shared by both ions (S1C and S1D Fig).

### Divalent cation dependent activity and stability of Hantaan EN

**Fig 4:**
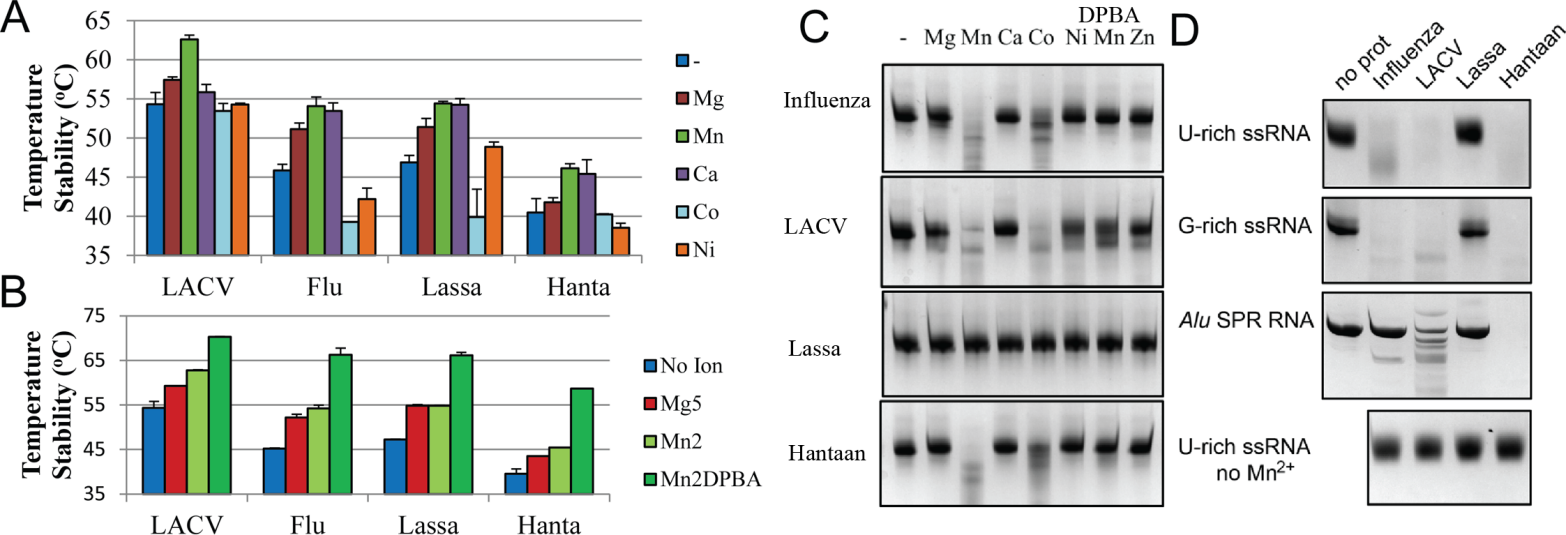
Comparative nuclease reaction and ion binding experiments for sNSV. **A**, Tm values derived from TSA experiments shown in bar diagrams for each EN in the presence of 2 mM of each indicated metal ion. **B**, Stabilization effects of 5 mM MgCl_2_ (Mg5), 2 mM MnCl_2_ (Mn2), and the super shift induced by 200 μM DPBA inhibitor (Mn2DPBA). **C,** Comparative nuclease activity assay of the indicated ENs in 2 h reactions at room temperature with G-rich RNA in the presence or absence of metal ions and DPBA inhibitor, all reactions were performed in parallel. **D**, Comparative nuclease activity assay with three distinct RNA substrates in 2 h reactions at room temperature, non-structured U-rich and G-rich RNA and highly structured *Alu* SRP RNA.

To study the ion binding specificity of Hantaan EN we measured the melting temperature (Tm) increase by a Thermal Shift Assay (TSA) in the presence of 2 mM of several metal ions (Fig 4A). This follows previous work showing that divalent metal ion binding increases EN thermal stability [10,14,24]. The Hantaan EN has a Tm of 40.5°C in the absence of metal ions. The Tm increases in the presence of MnCl_2_ (+5.6°C), CaCl_2_ (+4.9°C) and MgCl_2_ (+1.2°C), does not change in the presence of CoCl_2_ and is slightly lower with NiCl_2_ (-2°C). To define the specific ion binding preferences of Hantaan EN we compared, in parallel experiments, the stabilisation effect of metal ions on Lassa, LACV and IAV ENs (Fig 4A). The results show that Hantaan EN is generally the least stable, MnCl_2_ induces the highest stability increase for all ENs, MgCl_2_ also stabilise all ENs but less so for Hantaan EN and Ca^2+^ stabilizes Hantaan, IAV and Lassa ENs almost as much as Mn^2+^, but not LACV EN. The effects of CoCl_2_ and NiCl_2_ on Hantaan EN stabilization are similar to LACV and different to IAV and Lassa ENs (Fig 4A). Increase of the MgCl_2_ concentration from 2 mM to 5 mM further stabilises the Hantaan EN by 1.7°C (Fig 4B).Therefore, each EN has a specific ion stabilization pattern, but with the common feature that the highest Tm shift results from Mn^2+^ binding.

Subsequent TSA experiments were performed in the presence of 2 mM MnCl_2_ and 200 μM 2,4-dioxo-4-phenylbutanoic acid (DPBA) a known EN inhibitor [14,25] (Fig 4B). The addition of 2 mM Mn^2+^ and 200 μM DPBA induces a dramatic increase of the Hantaan EN stability (13.2°C), even larger than found for the other ENs tested (Fig 4B). DPBA has been shown to chelate the two metal ions in the active site in the homologous LACV and IAV endonucleases [14,26]. To confirm that Hantaan EN also binds two metal ions in solution, Isothermal Titration Calorimetry (ITC) experiments were performed to determine the binding affinities to Mn^2+^ and Mg^2+^. The Mn^2+^ binding profile could be fitted by a model that assumes sequential binding of two ions (S3A Fig). The first Mn^2+^ ion binds with a Kd_1_ of 48.5±2.6 μM and the second showed a much lower affinity with a Kd_2_ of 1.1±0.06 mM. The binding to Mg^2+^ is much weaker making it impossible to calculate reliable affinity values. The calculated affinities could be underestimated because of protein precipitation observed during the ITC experiment. However, these data are consistent with the two metal ion binding in the active site observed in the structure of the Hantaan EN and the stronger binding of Mn^2+^ ions compared to Mg^2+^.

We next investigated the ion dependence of the nuclease activity of Hantaan EN using IAV and LACV and Lassa ENs for comparison (Fig 4C). The reactions were carried out at room temperature for one hour with 2 mM of each metal ion, 7.5 μM of G-rich RNA and 10 μM protein. Hantaan EN shows clear nuclease activity with Mn^2+^ and also some with Co^2+^, but not with the other ions tested (Mg^2+^, Ca^2+^, Ni^2+^, and Zn^2+^). DPBA was able to inhibit the reaction with 2 mM MnCl_2_ at 200 μM. The control ENs, LACV and IAV, behaved as previously described [10,14,24] and showed a similar ion dependence to Hantaan EN. Surprisingly, under the same conditions Lassa EN showed no nuclease activity with any ion. In order to test the substrate specificity the same experiment was carried out in the presence of 2 mM MnCl_2_ on three different RNAs: unstructured U-rich and G-rich and the structured *Alu* RNAs (Fig 4D). The Hantaan EN is able to degrade all three RNAs efficiently showing no sequence or secondary structure specificity. Indeed Hantaan EN cleaves the structured *Alu* RNA more efficiently than LACV EN, which itself is more efficient than IAV EN (as previously reported [14]). Again, Lassa EN showed no activity against any RNA. In all cases no RNA degradation was detected in the absence of MnCl_2_ or in presence of 200 μM DPBA. The cleavage efficiency of the more structured RNA correlates with higher substrate accessibility to the active site (S2 Fig). The inhibitory effect of DPBA on Hantaan EN was further analysed by titrating increasing amounts of DBPA in EN assays with G-rich and *Alu* RNA in the presence of 2 mM Mn^2+^. LACV EN was used as a control. DPBA had a slightly higher inhibitory effect on Hantaan virus, with the IC50 estimated between 15 and 31 μM compared to the 62 μM estimated for LACV, in agreement with previously reported values for LACV (~50 μM, [14]) and with the TSA experiments where DPBA has a higher stability effect on Hantaan than on LACV EN (S4 Fig).

In conclusion, Hantaan EN is thermally stabilized most by Mn^2+^, consistent with it having the highest binding affinity for this ion. Mn^2+^ also most efficiently promotes the nuclease activity which is non sequence specific and inhibited by DPBA. The Hantaan EN efficiency for single stranded RNA appears similar to IAV and LACV but it is able to cleave structured RNA more efficiently than either IAV or LACV, probably due to the higher accessibility to the active site. Under the same experimental conditions, Lassa EN showed no nuclease activity at all.

### Analysis of Hantaan EN activity rates in comparison with LACV, IAV and Lassa ENs

**Fig 5:**
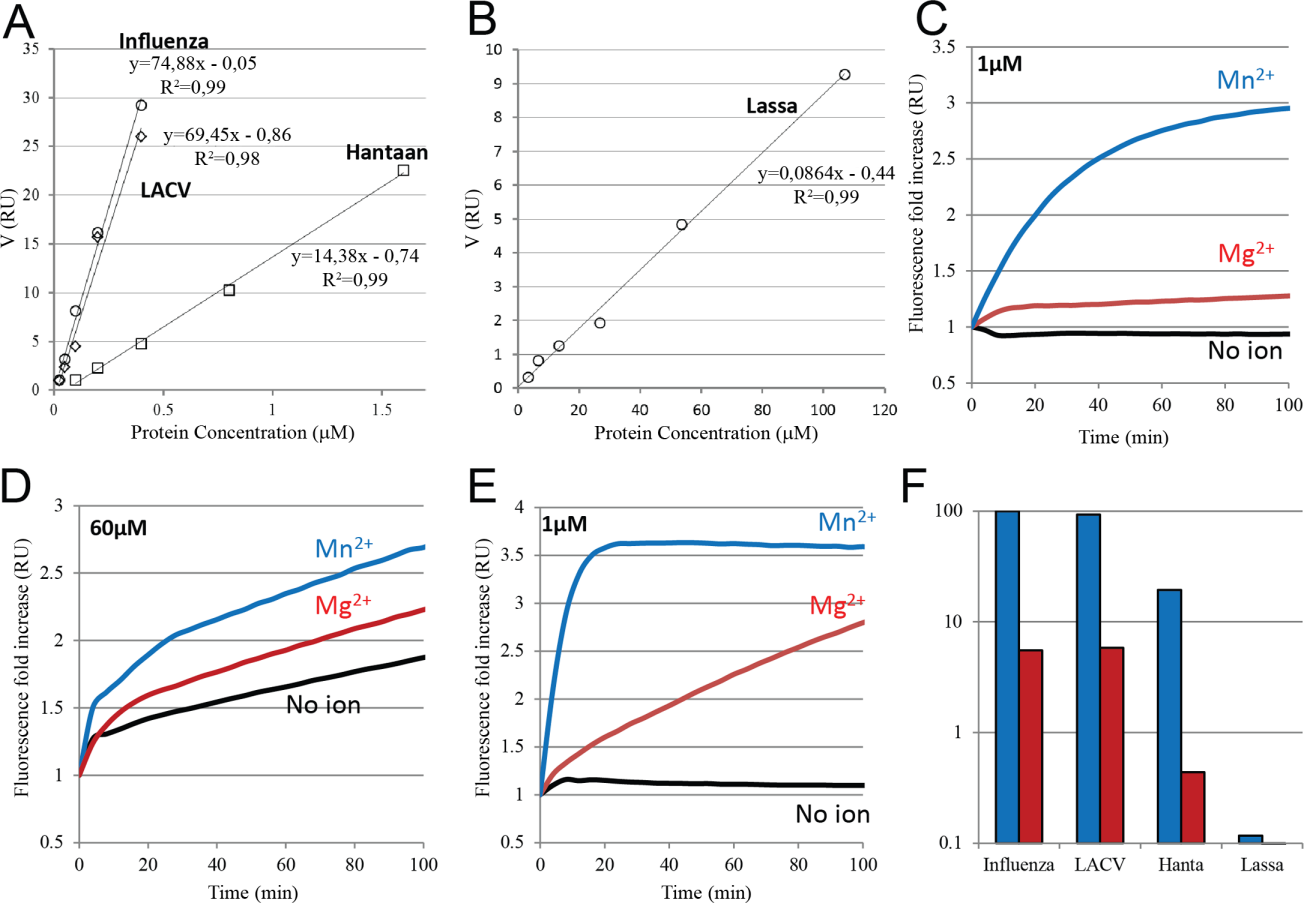
Fluorescent FRET based EN assays. Activity rates at increasing protein concentration of **A**, Influenza (circles), LACV (diamonds), Hantaan (squares) and **B**, Lassa (circles) ENs. The experiments were performed with 2 mM MnCl_2_ and 500 μM of fluorescent RNA. The velocity of fluorescent increase is plotted in the Y axes and the protein concentration in the X axes. **C**, Fluorescence based endonuclease assay curves. In red is shown the fold increase of Hantaan endonuclease at 1μM protein concentration with 2 mM MnCl_2_ (blue line) 5 mM MgCl_2_ (red line) and no ions (black line). **D**, Same experiment is shown for EN reactions with Lassa EN at 60μM protein concentration and **E,** for LACV EN at 1 μM protein concentration. **F,** A figure summary showing in logarithmic scale a bar diagram of panel A and B slopes values (blue bars) and the EN activity rates at 5 mM MgCl_2_ (red bars) from endonuclease experiments shown in panels C, D or E normalizing the data to IAV activity with Mn^2+^.

Intrigued by the differences in activity found between ENs from different families and genera we analysed nuclease activity rates with a more sensitive and quantitative real-time assay. This was done by measuring the fluorescence increase upon RNA substrate cleavage using a doubly labelled RNA (see Materials and Methods). For different protein concentrations, initial reaction velocities were determined as the initial slope of the reaction progression as monitored by the fluorescence signal. The linear relationship between the initial reaction velocity (V = ru/min, where “ru” is fluorescence relative units) and protein concentration is shown for Hantaan EN in Fig 5A. From the slope, a specific reaction rate of 14.38 ru min^-1^ μM^-1^ was derived. This activity is five-fold lower than for IAV or LACV ENs with rates of 74.88 and 69.45 ru min^-1^ μM^-1^ respectively (Fig 5A). The difference could be explained by the lower stability of the truncated Hantaan EN construct. On the other hand, the Hantaan EN is 180-fold more active than Lassa EN, which has a rate of 0.086 ru min^-1^ μM^-1^ (Fig 5B).

Since Lassa EN was reported to prefer Mg^2+^ as catalytic ion we also compared the activity with Hantaan, IAV, LACV and Lassa ENs in the presence of 5 mM MgCl_2_. Hantaan EN showed fifty-fold lower activity in the presence of 5 mM MgCl_2_ than with 2 mM MnCl_2_ at 1 μM of protein concentration and no activity was detected in the absence of metal ions (Fig 5C). IAV and LACV showed at the same protein concentration 20 fold slower activity with 5 mM MgCl_2_ than with 2 mM MnCl_2_ (Fig 5E). For 60 μM Lassa EN (compared to typically 1 μM for the other ENs), the activity did not change significantly in the presence of 5 mM MgCl_2_ and in the absence of metal ions. This suggests that the observed weak Lassa EN activity must be, at least partially, ion-independent, perhaps due to contaminants which become significant at very high protein concentrations (Fig 5D). Concerned by this lack of activity, we measured the ion binding to Lassa EN by ITC. Lassa EN showed two Mn^2+^ binding sites with Kd values of 21.2 ± 0.7 μM and 120.6 ± 4.7 μM. The interaction with Mg^2+^ gave a lower signal that could be fitted by a one site model with a much higher Kd of 352.0 ± 7.3 μM (S3B Fig).

In Fig 5F we summarise the quantitative nuclease activity results. Hantaan virus EN has between four and five fold less activity than LACV and IAV and Lassa virus 800 fold less activity in presence of 2 mM MnCl_2_. The activity drops to 6.5, 5.5 and 2.2% respectively for LACV, IAV and Hantaan upon substituting 2 mM MnCl_2_ by 5 mM MgCl_2_. We conclude that Hantaan, together with LACV and IAV ENs are active ENs that preferentially use Mn^2+^ but can also use Mg^2+^ with much lower activity rates. By comparison Lassa EN is virtually inactive in the presence of either Mg^2+^ or Mn^2+^ metal ions, even if it is able to bind them with similar affinities.

### Mutational analysis of the endonuclease activity and ion induced stability of Hantaan EN

**Fig 6:**
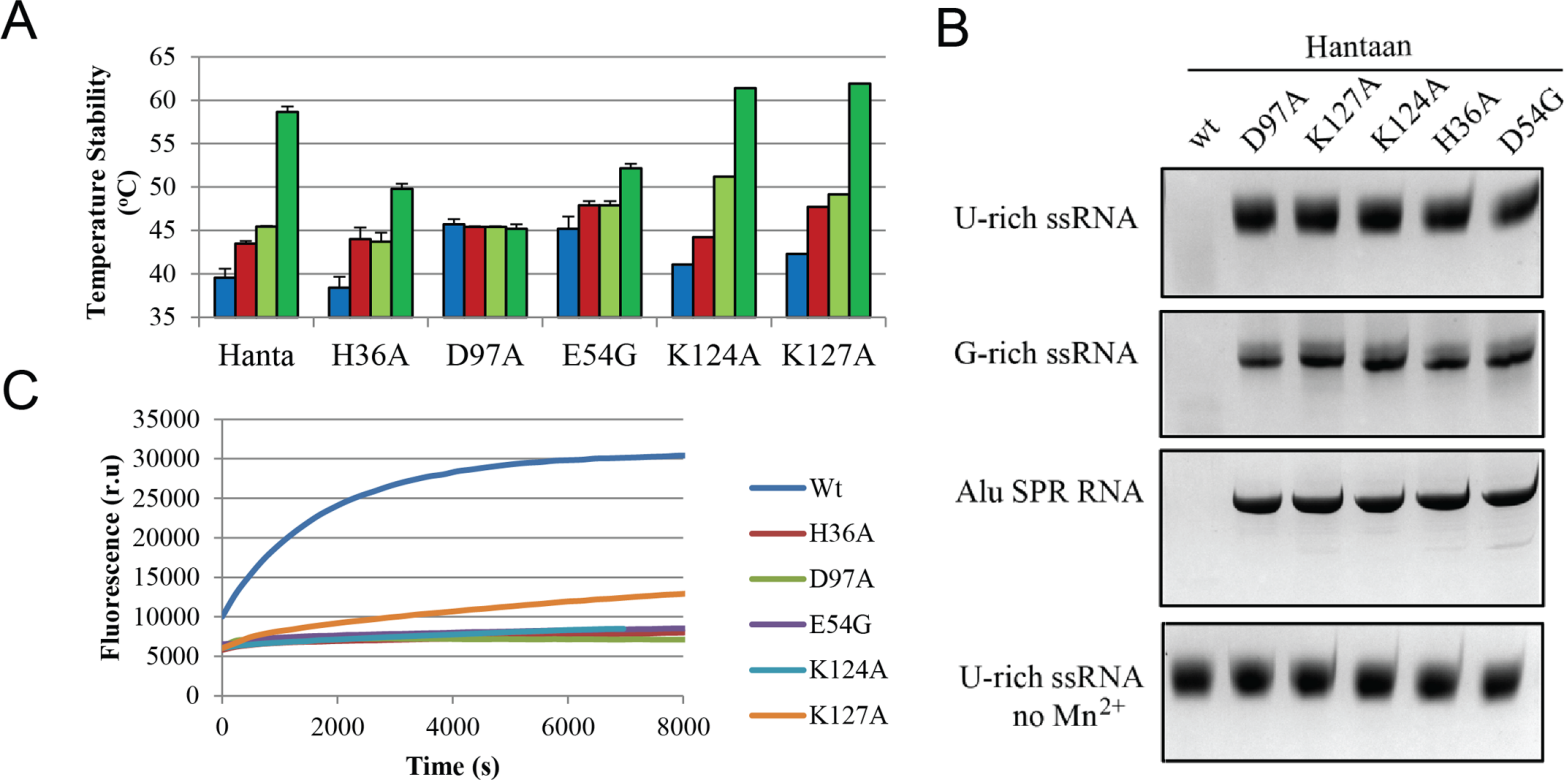
Mutational analysis of Hantaan EN *in vitro* EN activity and thermal stability. **A,** TSA experiments showing EN stabilization by metal ions and DPBA. Tm values (bars) are shown for mutated Hantaan ENs in comparison with the wild-type with either no ions, 5 mM MgCl_2_, 2 mM MnCl_2_ or 200 μM DPBA plus 2 mM MnCl_2_ and are coloured as in Fig 4B. **B,** Nuclease activity assays of Hantaan EN mutants with U-rich and G-rich RNAs and *Alu* SRP RNA in 2 h reactions at room temperature. **C,** Raw data representation of FRET based EN assays comparing the activity of Hantaan wild-type and mutated ENs at 1 μM protein concentration.

To elucidate the role for the Hantaan EN active site residues in ion binding and catalytic activity the mutants H36A, E54G, D97A, K124A and K127A were produced. We first analysed the effect of mutation on protein stability by TSA with either no ions, 5 mM MgCl_2_ or 2 mM MnCl_2_ with and without 200 μM DPBA (Fig 6A). In the absence of ions, removal of negatively charged sidechains (D97 and E54) resulted in a greater than 5°C protein stability increase, whereas removal of positive charges (H36, K124, K127) had a lower effect on stability changes. Mutation of H36 slightly reduced the Mn^2+^ and Mg^2+^ stabilization effect but reduced more DPBA stabilisation, to 50% of the wild-type. Mutation of the D97, which coordinates both ions, resulted in a complete lack of ion or DPBA stabilisation. Mutation of E54, in the flexible loop and coordinating Mn2, impairs both the ion stabilization effect and reduces the DPBA super shift. When the putative catalytic lysine K124 and its neighbour K127 were mutated to alanine the stabilization by ions and DPBA was not impaired and even slightly enhanced. These results are consistent with the crystal structure where H36, D97 and E54 are engaged in ion coordination but K124 and K127 are not. Subsequently, ITC experiments were performed with mutants E54G and D97A. E54G shows, instead of the wild-type two ion binding profile, a binding profile consistent with one Mn^2+^ binding site with a Kd = 387.6 ± 6.2 μM, higher than the wild-type for Mn1 binding (Kd = 48.5 ± 2.6μM, see above) whereas for D97A almost no binding was detected (S3C Fig). This confirms the loss of one binding site (Mn2) and both binding sites (Mn1 and Mn2) upon removal of respectively E54 and D97 side chains, in agreement with the ion coordination observed in the Hantaan EN crystal structure.

To determine the role of the mutated residues in the catalytic activity we carried out EN assays with 2 mM MnCl_2_ and the three different RNAs in parallel with the wild-type EN (Fig 6B). All mutations abolished Hantaan EN activity. The mutant’s activity was also tested by the more sensitive FRET based EN assay. Again, the mutants showed a dramatic drop of reaction rate compared to the wild-type. Only K127A showed clear EN activity above the other mutants, but still much lower than the wild-type (Fig 6C).

Therefore those active site conserved residues that are observed to coordinate the metal ions in the crystal structure are important in solution for both ion binding and endonuclease activity, as is the putative catalytic lysine K124. A neighbouring residue, K127, is also important for EN activity, possibly in substrate binding, but is not essential since its mutation still allows a low activity rate.

### Structural characterization of Mn^2+^ ion binding to Lassa EN

To investigate the cause of the lack of activity of Lassa EN we structurally characterized the Mn^2+^ ion bound form by X-ray crystallography to see how it differs from the active ENs. Using a construct encompassing residues 1–174 from Lassa L protein (see Materials and Methods) we obtained three different crystal forms: one with no ions bound (crystal form X1), and two with one or two Mn^2+^ ions bound in the active site (crystal forms X2 and X3 respectively).

**Fig 7:**
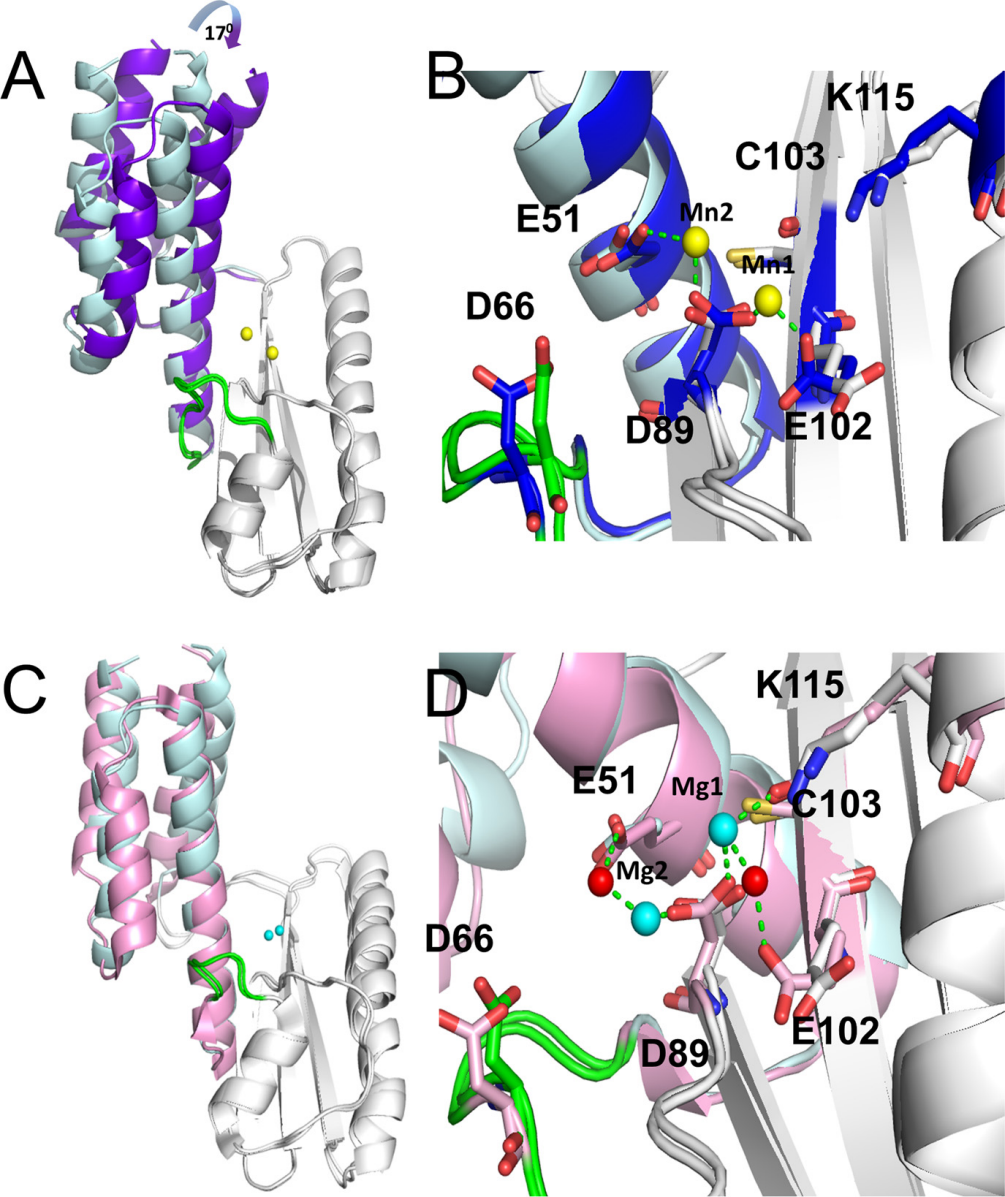
Lassa EN structures, flexibility and metal ion binding. **A,** Cartoon representation of the Lassa X1 and X3 structures after superposition. The α-helical bundles of X1 and X3 are respectively coloured in light and dark blue. The catalytic loop is highlighted in green and the Mn^2+^ ions of X3 are yellow spheres. The α-helical bundle closes the active site by the indicated rotation (blue arrow). The Lassa X2 structure has the same conformation as X3 (see S5A and S5B Fig). **B,** The same structure superposition as **A**, with the active site residues as sticks, the X3 Mn^2+^ metal ions as yellow spheres and the ion coordination indicated by dashed green lines. **C,** Comparison of the Lassa X1 structure (light blue) and the previously reported Lassa EN structure in complex with Mg^2+^ [16] (light pink, PDB: 4MIW). The Mg^2+^ ions are shown as cyan spheres. **D,** The same superposition as **C,** with the active site residues as sticks and the bridging water molecules as small red spheres. The ion coordination and hydrogen bond network is shown by green dashed lines.

The X1 structure was solved by molecular replacement using LCMV EN structure (PDB: 3JSB) and refined at 1.85 Å resolution (see Materials and Methods and Table 1). It is similar to the reported Lassa EN structure in complex with Mg^2+^ [16]. Despite the presence of 2 mM MnCl_2_ in the crystallization buffer no anomalous scattering was detected in the active site. The X2 data, solved by molecular replacement using the X1 structure, reached an ultra-high resolution of 1.09 Å, thus providing a highly accurate electron density map of the Lassa EN. Compared with the X1 form, the X2 and X3 forms show a 17 degree rotation between the helical bundle lobe (residues 4–49 and 149–167) and residues 50–148, with the hinge being at the base of helix αb (Fig 7A). The closure of the two lobes slightly changes the orientation of the active site residue E51 (see below and Fig 7B). In the X2 form, data measured close to the manganese absorption edge, showed a 50σ anomalous peak corresponding to one Mn^2+^ ion in the active. X3 was obtained by co-crystallization with the inhibitor DPBA in the presence of 5 mM MnCl_2_. The structure was solved from the X2 model and refined to 2.36 Å resolution. Whereas structures X2 and X3 both exhibit the closed form (S5A and S5B Fig), X3 has two Mn^2+^ ions in the active site (as detected by anomalous scattering, S5C Fig), but no extra density for DPBA.

Figs 1 and 2 compare the fold of the Lassa X3 structure with the other sNSV ENs. Lassa EN has the basic EN fold made by three beta strands (βa-c) and two alpha helices (αc-d) without any insertion. The helical bundle lobe, in comparison with the LACV EN, comprises four long α-helices, the N terminal αpre-a being additional. The active site residues are between the two lobes. The loop connecting αb and αc is also conserved, but instead of approaching the active site, as in Hantaan, LACV and IAV, it is turned outwards and the acidic residue D66, equivalent to the catalytic residues E80 (IAV) D52 (LACV) and E54 (Hantaan), is distanced from the active site (Fig 3D).

Both the X2 and X3 structures share a common Mn^2+^ site (Mn1) which is coordinated by the D89 and E102 side chains with 2 Å bond distances, but, unlike other ENs, the carbonyl oxygen of the C103 backbone is too distant for a direct interaction. In the X3 structure, a second Mn^2+^ ion (Mn2) is found in the active site coordinated by D89 and one carboxyl oxygen of E51 at 2.4 Å distance (Fig 3D). This two metal ion binding is additionally stabilised by crystal contacts with N-terminal residue E3 from the neighbouring symmetry related molecule also coordinating Mn2 (Fig 3D). The Mn^2+^ ion pair in Lassa EN is differently orientated with respect to the catalytic residues than in Hantaan, LACV and IAV ENs, and only four of the eight possible octahedral coordination bonds for the two Mn^2+^are satisfied (Fig 3). The previously reported Lassa EN structure in complex with Mg^2+^ ions [16] has an open conformation of the two lobes, similar to the X1 apo-structure (Fig 7C). The active site residues have the same rotamer conformation than in the X3 structure (Fig 7B and 7D) but the helix αb is more open, slightly enlarging the active site. The two Mg^2+^ ions are both directly coordinated by D89. Furthermore, Mg1 interacts directly with the C103 backbone carbonyl oxygen and indirectly with E51 and E102 by bridging water molecules. However the full canonical ion co-ordination observed in the active His+ ENs is not achieved.

The two Mn^2+^ ions binding mode reported here for Lassa EN is non-canonical when compared to the mode of ion binding in the His+ ENs. This is likely a consequence of several factors, including the replacement of the histidine by a glutamic acid (E51), the loss of one residue potentially coordinating the metal ions (D66, that is far from the active site) and the observed flexibility of the active site that is able to alter configuration because of the hinge at the base of helix αb. In comparison, Hantaan, LACV and IAV ENs have more constrained active site with higher ion coordination provided by an acidic residue from the flexible loop and the presence of a histidine that helps stabilise the ion binding in an active configuration.

### Mutational analysis of the endonuclease activity and ion induced stability Lassa EN

**Fig 8:**
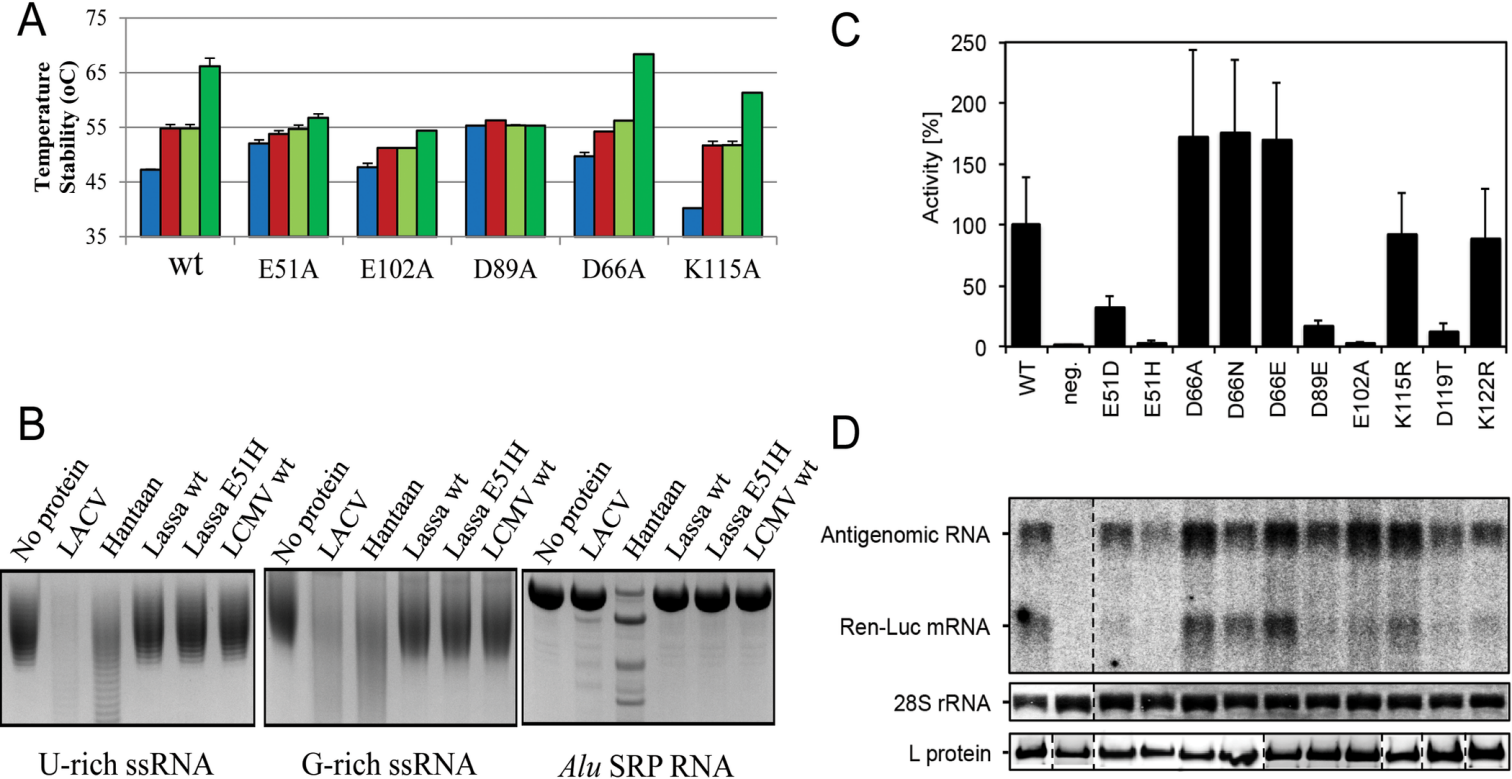
Mutational analysis of Lassa EN minireplicon transcriptional activity and thermal stability. **A,** TSA experiments showing the stabilization by metal ions and DPBA. Tm values (bars) are shown for mutated Lassa EN in comparison with the wild-type with no ions, 5 mM MgCl_2_, 2 mM MnCl_2_ or 200 μM DPBA plus 2 mM MnCl_2_, coloured as in Fig 4B. **B,** Nuclease activity assays of Lassa EN mutants in 1 h reactions at room temperature with U-rich and G-rich RNAs and *Alu* SRP RNA using LACV and Hantaan ENs as positive control. The LCMV EN (NL1 construct [13]), another His- EN, is also included in the experiment. **C,** Transcription and/or replication activity of L protein mutants was measured via Ren-Luc reporter gene expression. The Ren-Luc activity is shown in the bar graphs with mean standard deviation [n = 5] of standardized relative light units [sRLU] as a percentage of the wild-type (WT). A defective L protein with a mutation in the catalytic site of the RdRp served as a negative control (neg.). An activity less than 3% of the wild-type is considered as inactive, activities between 3 and 40% are intermediate and mutants with activity above 40% count as wild-type-like. **D,** Synthesis of antigenome and Ren-Luc mRNA was evaluated by Northern blotting using a radiolabelled riboprobe hybridizing to the Ren-Luc gene. As a marker for gel loading and RNA-transfer the methylene blue stained 28S rRNA is shown. Immunoblot bands of FLAG-tagged L protein mutants give proof of protein expression.

To further investigate these findings on Lassa EN, we mutated to alanine the amino acid residues E51, D89 and E102, which are engaged in ion coordination, D66 from the flexible loop and the putative catalytic lysine K115 (whose side-chain amide group superposes exactly with that of catalytic K137 in IAV EN). TSA experiments again show a stability increase, in the absence of ions, when active site acidic residues are mutated to alanine, presumably due to the loss of electrostatic repulsion between these residues (Fig 8A). The stability increase induced by metal ions and DPBA was severely impaired for the E51A mutation that removes one Mn2 coordination in the X3 Lassa EN structure, and for E102A, that removes one Mn1 coordination, and completely abolished for D89A, which removes the coordination with both Mn1 and Mn2 ions. Mutations D66A and K115A, which do not contact the catalytic ions in the X3 structure, did not significantly affect the ion stabilisation effect. For the D66A and D89A mutants, Mn^2+^ binding was tested by ITC. The D66A thermogram fits a two ion binding model with affinities of K_d_1 = 16.9 ± 0.6 μM and K_d_2 = 153.8 ± 62.3 μM, similar to the wild-type protein (K_d_1 = 21.2 ± 0.7 μM, K_d_2 = 120.6 ± 4.7 μM, see above). D89A resulted in a complete loss of ion binding (S3D Fig). Altogether this data is consistent with the non-canonical two ion binding of Lassa EN observed in the X3 crystal structure.

We also tested whether an E51H substitution could increase the activity of Lassa EN, by potential conversion of a His- to a His+ EN, but observed the same lack of endonuclease activity shown by the wild-type Lassa EN. LCMV, another His- EN, was included in this experiment showing that, in our experimental conditions and like Lassa EN, it lacks activity in comparison with the LACV and Hantaan ENs included in the experiment as positive controls (Fig 8B). Therefore changing the catalytic E51 to a histidine is not enough to confer efficient EN activity to Lassa EN.

Despite the fact that the isolated Lassa EN is almost inactive *in vitro*, the full length polymerase is clearly able to carry out cap-snatching dependent transcription in the cellular context. Indeed, using a minireplicon system, it has already been confirmed that active site residues D89, E102, D129 [17] and E51 and K115 [16] are essential for cap-dependent transcription. Because of the very low activity of the isolated Lassa EN *in vitro* we decided to test the activity of new mutants in the full length L protein using the same approach, with the E102A mutant as negative control (Fig 8C and 8D). D66 was mutated to alanine truncating the sidechain, to glutamic to extend the side chain by one carbon, and to asparagine for the removal of the negative charge. All mutants showed about a 50% increase of transcriptional activity confirming that this residue does not play an essential metal-binding role, consistent with the TSA and ITC experiments just described, but unlike the equivalent residues in Hantaan (E54), LACV (D52) and IAV (E80). Consistent with *in vitro* results, which showed the E51H mutant EN to be inactive (Fig 8B), we also found that in the full-length context this mutant did not support any transcription. Some conservative mutations were performed on the critical catalytic residues (E51D, D89E and K115R) and residues involved in hydrogen bonding close to the active site (K122R and D119T, which makes hydrogen bonds with both K115 and K122). The mutations of the acidic residues showed a certain drop of transcriptional activity, but not as much as the E102A mutation negative control. Interestingly both lysine to arginine mutations did not change the transcriptional activity.

Based on these results we can conclude that the acidic residue on the flexible loop (D66) in Lassa EN is not essential for activity (as it is in the His+ ENs), D51 is not replaceable by histidine, and that even if conservative mutations in the putative catalytic K115 or neighbouring residues (K122 and D119T) do not significantly change the transcriptional activity yields, these residues seems to be important in natural infection as shown by their conservation in sequence alignments of arenavirus ENs (S6 Fig).

## Discussion

The cap-snatching mechanism for transcription is exclusively used by sNSVs. The partial picture provided by the isolated endonucleases and cap-binding domain structures has been dramatically extended recently with the structure determination and biochemical characterization of the heterotrimeric influenza polymerase and the major part of the LACV L protein, allowing the understanding of how the cap-snatching EN domains integrate with the RdRpol domain [9,12,27–30]. Furthermore, in influenza virus polymerase, the EN domain (as well as several other PB2 domains including the cap-binding domain) is connected to the polymerase core through a flexible hinge, which allows it to adopt multiple conformations including those competent for cap-snatching and others where access to the cap-snatching domains is hindered [28,30]. 5' vRNA end binding is required to induce the correct relative positioning of the cap-binding and cap-snatching endonuclease domains to increase the RNA cleavage efficiency by 100-fold compared to the isolated EN domain [30]. This allows, for instance, an efficient EN activity with MgCl_2_ in the context of the full length polymerase that is not achievable by the isolated domain [9]. This shows that the activity of the cap-snatching endonuclease can be significantly modified in the context of the full length polymerase and this is likely to be also the case in the arena- and bunyavirus L proteins.

Here we demonstrate that hantavirus L proteins also have a cap-snatching endonuclease that shares with LACV bunyavirus and IAV the same configuration of the active site and contributes to define the canonical binding of metal ions in the active sites by which all the catalytic residues of the H.PD.E/D motif are directly coordinating the two metal ions oriented towards the catalytic lysine (Fig 3). The mutation of any of these residues affects the ion binding and, results in the complete loss of catalytic activity, as does mutation of the catalytic lysine. To be able to express the Hantaan EN we had to delete part of its C-terminus, which may impair the activity to some extent, although the measured activity rate is comparable to those of LACV and IAV. Indeed, the wild-type Hantaan EN activity could be much higher, resulting in toxicity for *E*.*coli* cells, as observed in the accompanying article ([22], co-submitted) for the full length wild-type Andes virus EN, where only reducing the activity by mutagenesis near the active site made expression possible.

In the case of arenavirus ENs the most significant active site differences correspond to Lassa EN E51, that substitutes the bunya- and orthomyxovirus conserved histidine, and D66, which is not any more engaged in the active site, unlike the equivalent acidic residue in the bunya- and orthomyxovirus EN flexible loop. This study shows that in Lassa EN these differences result in the non-canonical binding of metal ions to the isolated enzyme causing a dramatic drop of endonuclease activity in comparison with the His+ ENs. However the Lassa EN PD-D/E-K catalytic residues are essential for transcription in the minireplicon context, showing that in the full length polymerase the nuclease efficiently cleaves cellular mRNA. Since arenavirus EN active site is more open than His+ ENs the catalytic residues cannot directly coordinate the two metal ions in the same way. Even in the Lassa EN X3 crystal form, were the active site is more closed than X1 form, the open helix αb conformation maintains E51 2 Å away from the Mn1 coordination site (S5D Fig). Therefore we speculate that to achieve the canonical binding required for activity, the Lassa active site needs to be activated for instance by changing E51 and E102 rotamers coupled to a slight movement of helix αb that would close the active site allowing the coordination of Mn1 as shown by the active His+ ENs (S7 Fig). Another possibility is that residues from other parts of the polymerase might contribute to the ion coordination. These changes could be induced by other parts of the L-protein in response for example to vRNA binding (as in IAV) or induced by substrate binding. The shift between an active or an inactive enzyme would provide arenaviruses with an “on and off” transcription switch.

With the addition of the Hantaan EN structure and new results of Lassa EN, this comparative study puts previously reported work on the isolated cap-snatching endonucleases from bunyavirus, orthomyxovirus and arenavirus in a more general context. We find two different kinds of endonucleases, one with the characteristic catalytic histidine (His+) as in orthomyxovirus and bunyavirus, which have efficient endonuclease activity in isolation, and a second, without the histidine (His-) and conserved among arenaviruses which shows very poor activity *in vitro*. This classification should be taken into account in further development of inhibitor screening assays targeting sNSV ENs. Furthermore, the structure of the active Hantaan EN provides another tool towards the comprehensive development of broad spectrum antivirals against sNSV.

## Materials and Methods

### Cloning and mutagenesis

*E*. *coli* codon optimised coding sequences were synthesised (Geneart) for residues 1–250 of Lassa polymerase (Lassa 250) and residues 1–182 of Hantaan polymerase (Hanta 182) (UniProt accession code Q6GWS6 and P23456 respectively). A histidine tag and a Tobacco Etch Virus (TEV) cleavage site (MGHHHHHHDYDIPTTENLYFQG-) were added to the amino terminus of all protein constructs. For the final Hantaan constructs a SUMO tag was inserted between the histidine tag and the TEV cleavage site. All protein variants were cloned into a modified pET9a (Novagen) vector as described [14]. Mutagenesis of the proteins expressed in *E*. *coli* was performed on Lassa196 and Hanta182. Mutant constructs were obtained by site directed mutagenesis using overlapping oligonucleotides and Pfu DNA polymerase.

### Protein expression and purification

Proteins were expressed in *Escherichia coli* strain BL21 (DE3) in LB media with 25 mM kanamycin at 18°C overnight after induction with 0.2 mM of IPTG. The protein was purified as previously described, removing the histidine or His-SUMO tags by TEV protease resulting in an additional glycine before the first translated methionine of the original sequence. The resulting untagged proteins were concentrated and purified by gel filtration chromatography using a SD75 column (Pharmacia) with lysis buffer (20 mM Tris-HCl pH 8.0, 150 mM NaCl, 5 mM β-mercaptoethanol) for *in vitro* experiments and crystallization trials. Hantaan required 1mM TCEP in the lysis buffer to avoid aggregation. IAV A/H1N1 EN (PA residues 1–198) and La Crosse EN (LACV-L 1–183) were expressed as described elsewhere [26] [14] and purified as Lassa and Hantaan ENs.

### Identification of the amino terminal EN domain of Lassa and Hantaan L-proteins

Several protein constructs including the first 179 to 250 aa of the Hantaan L protein N-terminus were tested for expression in *E*.*coli*. Only the shorter constructs (179–185 aa) were expressed, but insolubly into inclusion bodies. This could be circumvented by insertion of an N-terminal SUMO tag linked by a TEV cleavage site.

The length of the proteolytically stable amino terminal domain was defined from the purified Lassa 250 protein by limited papain digestion with 1:500 (w:w) papain: protein ratio. Products were characterized by N-terminal sequencing (Edmann degradation) and mass spectrometry (Electrospray). Two papain resistant fragments were obtained with molecular weights of 22.0–22.8 kDa and 25.4 kDa corresponding to the first 191–198 or 222 residues respectively of the Lassa L-protein. Proteins Lassa 190, 192, 196, 205, 221 were subsequently produced for crystallization trials. Based on the first crystal structure obtained, the constructs Lassa 174, 177, 180, 186 were cloned. Finally, the protein construct Lassa 196 was used for *in vitro* biochemical experiments and Lassa 174 for structural studies.

### Thermal stability experiments and EN activity assays

The influence of manganese, magnesium and DPBA binding on protein stability was measured by thermal stability assays (TSA) [31] at a protein concentration of 7.5 μM in lysis buffer implemented with 2 mM or 5 mM metal ion concentration or 2 mM Mn^2+^ plus 200 μM DPBA concentration. For nuclease activity experiments, 10 μM of Influenza-PA 1–209, LACV-L 1–183, Lassa 1–196 and Hantaan 1–182 wild-type and mutant proteins were incubated with 10 μM of *Alu* RNA (110 nucleotides of the *Alu* domain of *Pyrococcus horikoshii* SRP RNA) or 15 μM of 44 nucleotides U-rich (5’-GGGCCAUCCU GCUCU_4_CCCU_11_CU_11_-3’) and G-rich (5’-GGGCCAGGAAAGGGAGGAGA AAG_11_AAAAGG AGAAA-3’) RNAs for 1 or 2 h at room temperature in the same buffer. The metal ion concentration was 2 mM. The reaction was stopped by adding loading buffer, 10 M urea and twofold concentrated Tris-borate-EDTA buffer (TBE). The reaction products were loaded onto 15% acrylamide 8 M urea TBE gels and stained with methylene blue.

For the FRET based real-time quantitative endonuclease activity assays 500 nM of synthetic double labelled RNA, 6-FAM-5′-CUCCUCAUUUUUCCCUAGUU-3′-BHQ1 (IBA), were mixed with the endonuclease proteins. The reaction buffer was 20 mM Tris-HCl pH 8, 150 mM NaCl, 1 mM TCEP and 2 mM MnCl2 or 5 mM MgCl_2_. The fluorescence increase upon the RNA cleavage was measured in a TECAN (infinite M200 pro) at 26°C using 465 nm excitation and 520 nm emission wavelengths. The initial reactions velocities were determined by the slope of the linear part of the reaction and where the fitting quality for a straight line was above R^2^ = 0.99.

### Isothermal titration calorimetry (ITC)

ITC measurements were performed at 25°C, using an ITC200 Micro-calorimeter (MicroCal, Inc). Experiments comprised 26 injections of 1.5 μL of 2.5–5.0 mM manganese or magnesium solutions into the sample cell containing 200 μL of 100–160 μM of Lassa EN (wild-type, E51A or D89A) or Hantaan EN (wild-type, E54G or D97A). All binding studies were performed in the lysis buffer. For data analysis the heat produced by the metal ion dilution in the buffer was subtracted from the heat obtained in the presence of the protein. Binding isotherms were fitted to a one-site binding or two-site sequential binding model using Origin Software version 7.0 (MicroCal, Inc). The initial data point was routinely deleted.

### Crystallization

Hantaan EN crystals were obtained with the NH179 and NH182 constructs at 10 mg/ml in lysis buffer with 2 mM MnCl_2_ mixed at 1:1 ratio with mother buffer Hepes 0.1 M pH7, 20% PEG 6K, 1 M LiCl_2_. The crystals grow at 20 ^0^C after 24 h and were frozen with mother buffer plus 30% glycerol and 2 mM MnCl_2_.

LACV and IAV were expressed and purified as previously described [14] [26]. Protein constructs denoted Lassa 174, 177, 180, 186,190, 192, 196, 205, 221 were expressed and screened for crystallization using a Cartesian robotic system [32]. Only Lassa 174 and Lassa 190 crystallised and Lassa 174 was used for all following work. The first crystals (X1) were obtained by mixing 1:1 ratio protein: reservoir solution of 5 mg/ml Lassa174 protein in lysis buffer with 2 mM MnCl_2_, and a reservoir composition of sodium citrate 0.191 M, PEG 3350 4%, 0.1 M Hepes pH7 and 0.1 M strontium chloride. Form X2 crystals were obtained with Lassa 174 in lysis buffer with 10 mM GMP and 2 mM MnCl_2_ and a reservoir composition of 0.1 M MES pH6 and 20% v/v 2-Methyl-2.4-pentanediol. Form X3 was crystallized with Lassa 174 in lysis buffer with 5 mM DPBA and 2 mM MnCl_2_ and a reservoir composition of 0.005 M magnesium chloride hexahydrate, 0.05 M HEPES-Na pH7 and 25% (v/v) PEG MME 550. All crystals grow at 20°C overnight. The crystals were frozen in liquid nitrogen in the reservoir buffer with 30% glycerol after adding 5 mM MnCl_2_ and 10 mM of GMP or 5 mM of DPBA for the co-crystallization derived crystals.

### Crystallography

The Hantaan EN crystals are of monoclinic space group (*P*2_1_), with two molecules in the asymmetric unit, and the structure was solved by a SAD experiment performed at 1.77 Å wavelength on beamline ID23-1 (ESRF) with high redundancy (Table 1). The data were processed with XDS and solved with CRANK [33] within the CCP4i package. The three Mn^2+^ ions and Met94 of each molecule gave the eight anomalous sites enabling structure solution. The structure was refined with a native dataset to Rwork/Rfree of 0.166/0.216 at 1.7 Å resolution obtained at 0.984 Å wavelen sequence alignment sequence alignment gth on ID23-1 with NH182 crystals (Table 1). Molecule A in the asymmetric unit shows density for residues 1–179, whereas molecule B shows residues 1–171 with a gap between residues 165 to 167.

Lassa EN form X1 crystals are of space-group *P*2_1_2_1_2_1_ with two molecules in the asymmetric unit. Data were collected on ID14-4 at the European Synchrotron Radiation Facility (ESRF) to 1.8 Å resolution using a wavelength of 0.939 Å. Data were processed and scaled with the XDS package [34] and subsequent analysis performed with the CCP4i package. Statistics of data collection and refinement are given in Table 1. The structure was solved by molecular replacement using Phaser and the LCMV EN model (PDB: 3jsb) split into two parts (residues 1–60 plus 147–170 and residues 61–145). The resultant map was excellent and could be largely built automatically by ARP/wARP [35]. The structure was refined to Rwork/Rfree of 0.205/0.259 at 1.85 Å resolution using REFMAC [36] (Table 1).

Lassa EN crystal forms X2 and X3 were obtained by co-crystallization with GMP and DPBA respectively. They are of space-group *P*4_1_2_1_2 with one molecule in the asymmetric unit. Two X2 datasets were collected on ID23-1 to 1.09 Å resolution, one at a wavelength of 0.976 Å and another close to the manganese edge (1.892 Å). Data were processed, scaled and the structure solved by molecular replacement using the X1 structure, which only succeeded after searching separately for the two lobes of the EN (residues 1 to 60 and 145 to 170, including most of the alpha helical bundle, and residues 65 to 140 including the β-sheet core of the protein). Data were refined to Rwork/Rfree 0.161/0.179 at 1.09 Å resolution (Table 1). Extra density for manganese is observed in the X2 active site, with a peak > 50 σ in the anomalous difference map calculated from the data collected at the Mn^2+^ edge. The X3 data were collected at SOLEIL on beamline PROXIMA1 to 2.5 Å resolution at a wavelength of 0.976 Å. The structure was solved from the X2 model and refined to Rwork/Rfree of 0.204/0.288 at 2.36 Å resolution. Extra density for two manganese ions is observed in the active site, consistent with the anomalous difference map. A third Mn^2+^ also is found bound to His75 on the protein surface. There is no extra density consistent with DPBA or GMP in the active sites of the X2 or X3 structures.

### Evaluation of endonuclease activity using the Lassa virus replicon system

The T7 RNA polymerase-based Lassa virus replicon system was used as described previously [37,38] [17]. Briefly, generation of L genes with single mutations in the endonuclease domain was performed by mutagenic PCR using pCITE-L as a template and Q5 High-Fidelity DNA Polymerase (NEB) for amplification. After purification and spectrophotomeric quantification the PCR-products were directly used for transfection. To make sure the specific mutations were present the PCR-products were sent for sequencing. For Luciferase measurements and RNA extractions BSR-T7/5 cells stably expressing T7 RNA polymerase (kindly provided by Ursula Buchholz and Karl-Klaus Conzelmann) [39] were transfected in a 24 well plate with the following amounts of DNA per well: (a) 250 ng of minigenome expressing Renilla luciferase (Ren-Luc), (b) 250 ng of L gene PCR-product, (c) 250 ng of pCITE-NP expressing NP, as well as (d) 10 ng of pCITE-FF-Luc expressing firefly luciferase (FF-Luc) as an internal transfection control. About 24 hours after transfection, either total cellular RNA was purified for Northern blotting using an RNeasy minikit (Qiagen) or cells were lysed and assayed for FF-Luc and Ren-Luc activity using the dual-luciferase reporter assay system (Promega). To compensate for differences in transfection efficiency and cell density Ren-Luc levels were corrected with the FF-Luc levels resulting in standardized relative light units [sRLU]. For Northern blot analysis, 500 ng of total cellular RNA was separated in a 1.5% agarose-formaldehyde gel and transferred onto a HybondN+ membrane (GE Healthcare). After UV crosslinking and methylene blue staining to visualize 28S rRNA the blots were hybridized with a 32P-labeled riboprobe targeting the Ren-Luc gene. Transcripts of Ren-Luc genes and RNA-replicates of the minigenome were visualized by autoradiography using an FLA-7000 phosphorimager (Fujifilm). To provide proof for expression of L protein mutants in BSR-T7/5 cells the cells were transfected with 500 ng of PCR product expressing C-terminally 3xFLAG-tagged L protein mutants per well in a 24-well plate. To boost the expression levels and thus facilitate detection by immunoblotting cells were additionally infected with modified vaccinia virus Ankara expressing T7 RNA polymerase (MVA-T7) [40]. After cell lysis and electrophoretic separation in a 3 to 8% Tris-acetate polyacrylamide gel proteins were transferred to a nitrocellulose membrane (GE Healthcare), and FLAG-tagged L protein mutants were detected using peroxidase-conjugated anti-FLAG M2 antibody (1:10,000) (A8592; Sigma-Aldrich). Detected bands were visualized by chemiluminescence using Super Signal West Femto substrate (Thermo Scientific) and a FUSION SL image acquisition system (Vilber Lourmat).

### Data deposition

The structure factors and PDB models are deposited in the PDB database as PDB: 5IZE for Hantaan EN, PDB: 5IZH for Lassa X1, PDB: 5J1N for Lassa X2 and PDB: 5J1P for Lassa X3.

## Supporting Information

S1 Fig Hantaan EN structure details on Mn^2+^ mediated crystal contacts, missing C-terminal alpha helices and active site configuration. **A,** Crystal contacts of Hantaan EN helix αa (in green cartoon) and a symmetry related molecule (blue cartoon), the Mn^2+^ is shown as a yellow sphere and the coordinating residues as sticks. The coordination is shown by dashed yellow lines. The octahedral coordination is completed by two water molecules shown in red small spheres. **B,** Superposition of the LACV EN structure (grey and purple cartoon) with Hantaan EN (pink cartoon) on the beta/alpha lobe. The Hantaan extra C-terminal beta strand is highlighted in yellow (Hantaan res 173–179) and the equivalent extended region of LACV structure is highlighted in purple (LACV res 142–183). **C,** The anomalous signal is shown for the two Mn^2+^ ions at 3 sigma using pymol. The octahedral coordination of the catalytic metal ions is shown in green dashed lines with the catalytic residues and water molecules. **D,** The active site of Hantaan EN is shown in grey cartoons with the catalytic residues as sticks, the Mn^2+^ as yellow spheres and waters as small red spheres. The 2Fo-Fc calculated density map is represented in blue at 1.5 sigma using Pymol.
(TIF)

S2 Fig Cap-snatching ENs active site accessibility. Surface representation of the ENs structures for Hantaan (wheat), Lassa (green), LACV (light blue) and Influenza (purple). The active site residues are coloured in yellow and the metal ions in red. A frontal and a side view of the active site shows the accessibility of the substrate to the active sites.
(TIF)

S3 Fig ITC experiments of the Mn^2+^ and Mg^2+^ binding to the Hantaan and Lassa ENs. Isothermal titration calorimetry measurements.(A) manganese (left) and magnesium (right) binding to Hantaan EN wild-type (B) Manganese binding to the Hantaan EN mutants E54G (left) and D97A (right). (C) Manganese (left) and magnesium (right) binding to Lassa EN wild-type. (D) Manganese binding to the Lassa EN mutants D66A (left) and D89A (right). I all cases the upper plot shows the binding isotherm and the lower plot shoes the integrated values of each corresponding isotherm after subtracting the heat produced by the metal ion dilution. The calculated affinity values are indicated for two ion binding (K_d_1 and K_d_2) or one ion binding model fitting (K_d_) and the fitting R^2^ values are indicated for each experiment. The red points were not used for the curve fitting. We used N = 1 for the one ion binding fits.
(TIF)

S4 Fig DPBA inhibitory effect on Hantaan and LACV ENs. **A,** Denaturant Urea-acrylamide gels of nuclease reactions with 2mM MnCl_2_ that were carried out in the presence of increasing concentrations of DPBA for Hantaan and LACV ENs in parallel. The experiment was performed with G-rich (upper panel) and *Alu* SPR RNA (bottom panel). The IC50 estimated concentration is indicated with a black diamond. **B,** The same experiment was performed in parallel with LACV EN.
(TIF)

S5 Fig Lassa EN structures, flexibility and metal ion binding. **A,** Superposition of Lassa X2 (grey cartoon) and X3 (purple cartoon) showing they have the same conformation. The manganese ions are represented as orange and yellow small spheres for the X2 and X3 respectively. **B,** Shows the same superposition as in **A** but focusing on the active site. The catalytic residues are represented by sticks and the Mn^2+^ ions by spheres as in **A** with the ion coordination indicated by dashed green lines. **C,** active site of the Lassa X3 structure shown as in Fig 3 with the anomalous difference density for X2 (purple mesh, at 50 σ) and X3 (orange mesh, at 3 σ). **D,** Superposition of Lassa X3 (light grey with catalytic residues as sticks) and LACV endonuclease structures (PDB: 2xi7) showing the LACV canonical position of Mn^2+^ ions and the catalytic histidine, highlighting the distance that E51 needs to move if it were to coordinate Mn1 as achieved by the catalytic histidine present in His+ ENs.
(TIF)

S6 Fig Sequence alignment of arenavirus ENs. Arenavirus ENs sequence alignment indicating the catalytic residues analysed by mutagenesis in the minireplicon system. The alignment shows that D66 is not conserved as the rest of residues.
(TIF)

S7 Fig Structural superposition of the active sites from the Lassa X1 crystal and the proposed model for Lassa EN activation. The structures of Lassa EN X1 (wheat) and the proposed model of active EN (light grey) are shown in cartoon with the active site residues shown in sticks. The two shown Mn^2+^ ions adopt the canonical binding like His+ endonuclease. This could be achieved by closing the active site by changing the E102 and E51 rotamers and slightly moving helix αb towards Mn1 position.
(TIF)

S1 Table rmsd pairwise values, and number of equivalent positions in parenthesis, calculated from pairwise structural alignments using Dalilite with the ENs used in this study: LACV (2XI7), Influenza H1N1 (4AVQ), LassaX3 and Hantaan EN structures. The molecule in the asymmetric unit used in the alignment is indicated in parenthesis for each PDB.
(PDF)
