# Supplementary figures and images for: Comparative Structural and Functional Analysis of Bunyavirus and Arenavirus Cap-Snatching Endonucleases
Source: PLoS Pathog. 2016 Jun 15;12(6):e1005636. doi: 10.1371/journal.ppat.1005636 (PMC4909276; doi:10.1371/journal.ppat.1005636)

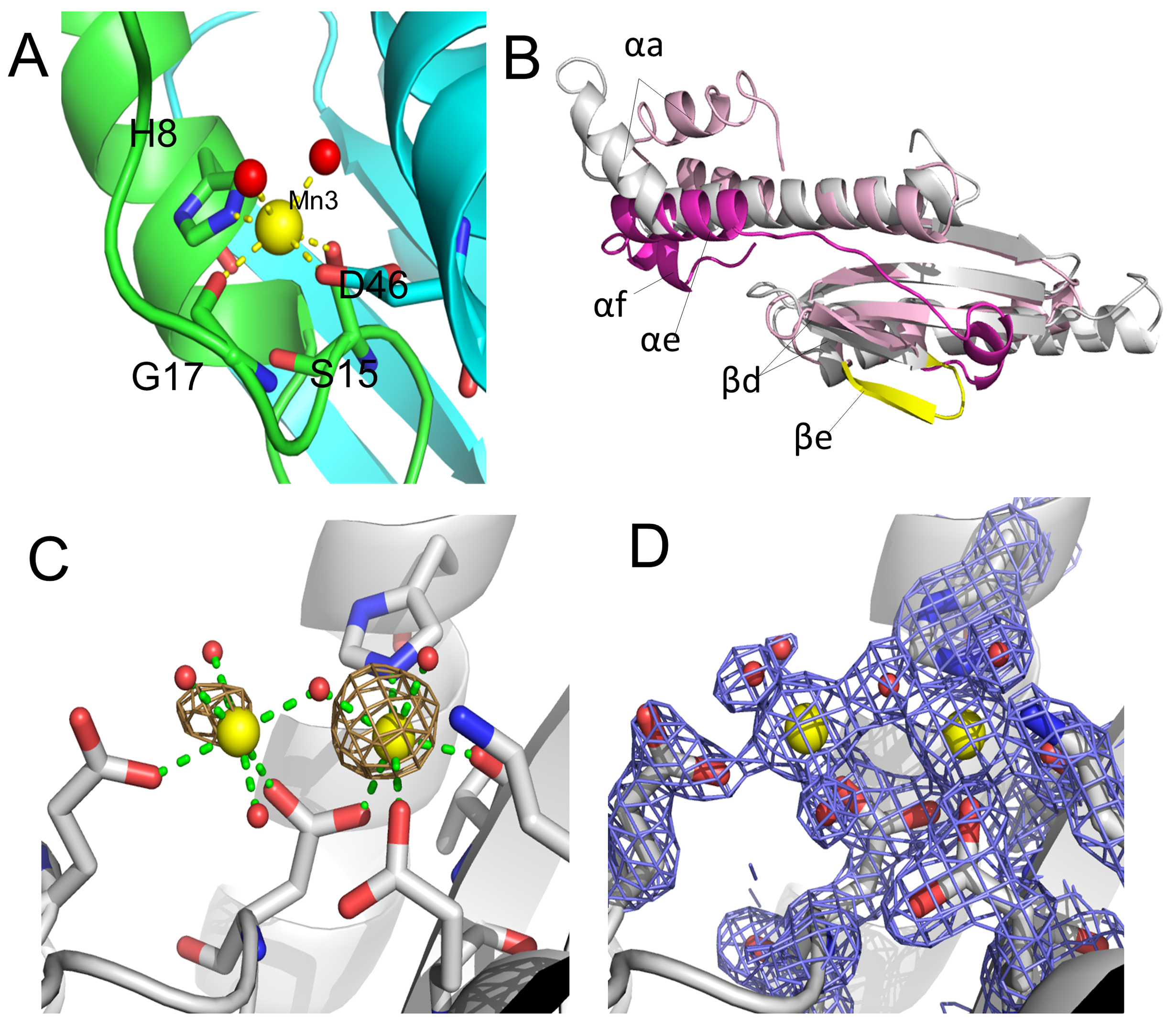

Supplement: S1 Fig — A, Crystal contacts of Hantaan EN helix αa (in green cartoon) and a symmetry related molecule (blue cartoon), the Mn2+ is shown as a yellow sphere and the coordinating residues as sticks. The coordination is shown by dashed yellow lines. The octahedral coordination is completed by two water molecules shown in red small spheres. B, Superposition of the LACV EN structure (grey and purple cartoon) with Hantaan EN (pink cartoon) on the beta/alpha lobe. The Hantaan extra C-terminal beta strand is highlighted in yellow (Hantaan res 173–179) and the equivalent extended region of LACV structure is highlighted in purple (LACV res 142–183). C, The anomalous signal is shown for the two Mn2+ ions at 3 sigma using pymol. The octahedral coordination of the catalytic metal ions is shown in green dashed lines with the catalytic residues and water molecules. D, The active site of Hantaan EN is shown in grey cartoons with the catalytic residues as sticks, the Mn2+ as yellow spheres and waters as small red spheres. The 2Fo-Fc calculated density map is represented in blue at 1.5 sigma using Pymol. (TIF) [file ppat.1005636.s001.tif]

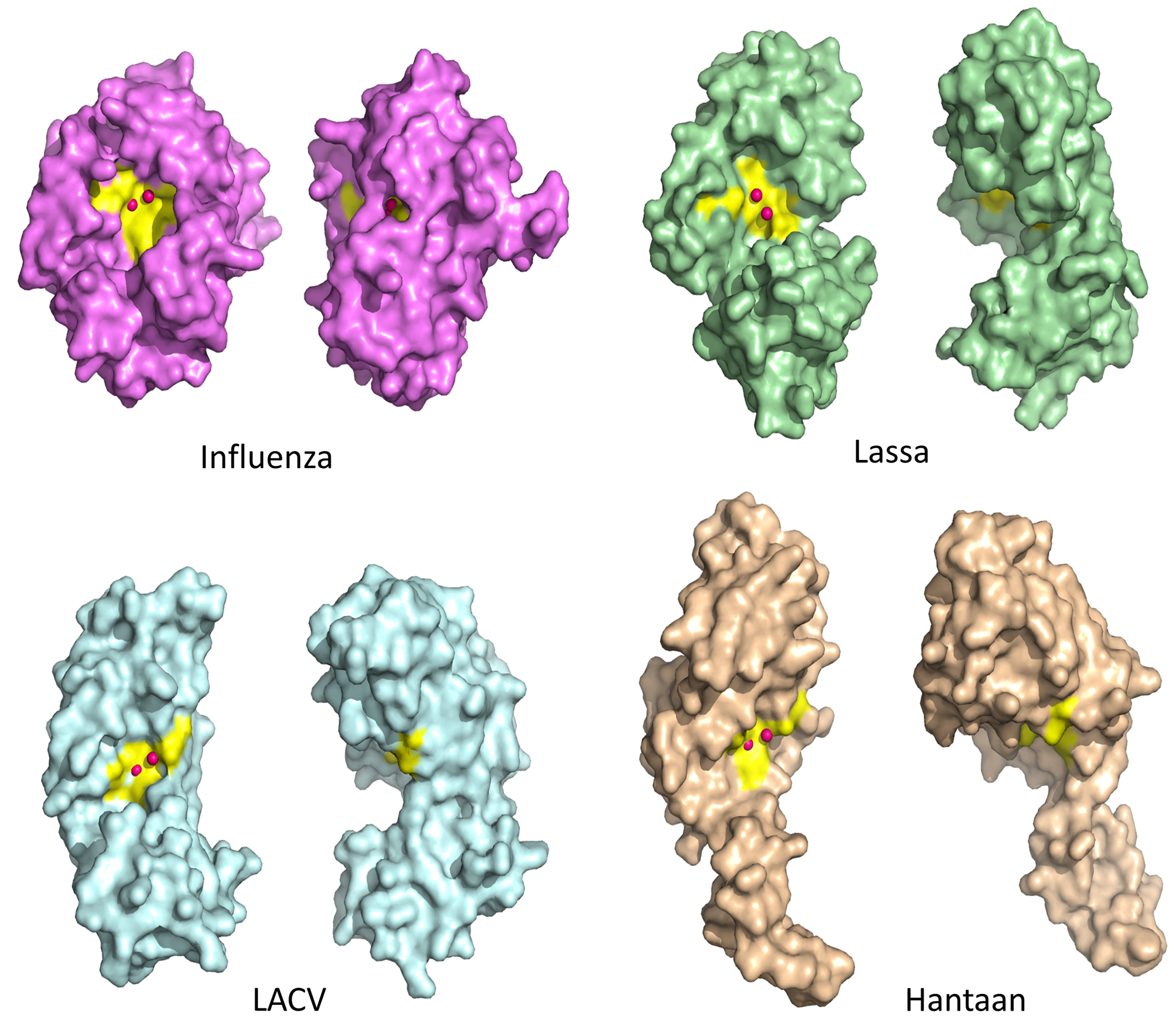

Supplement: S2 Fig — Surface representation of the ENs structures for Hantaan (wheat), Lassa (green), LACV (light blue) and Influenza (purple). The active site residues are coloured in yellow and the metal ions in red. A frontal and a side view of the active site shows the accessibility of the substrate to the active sites. (TIF) [file ppat.1005636.s002.tif]

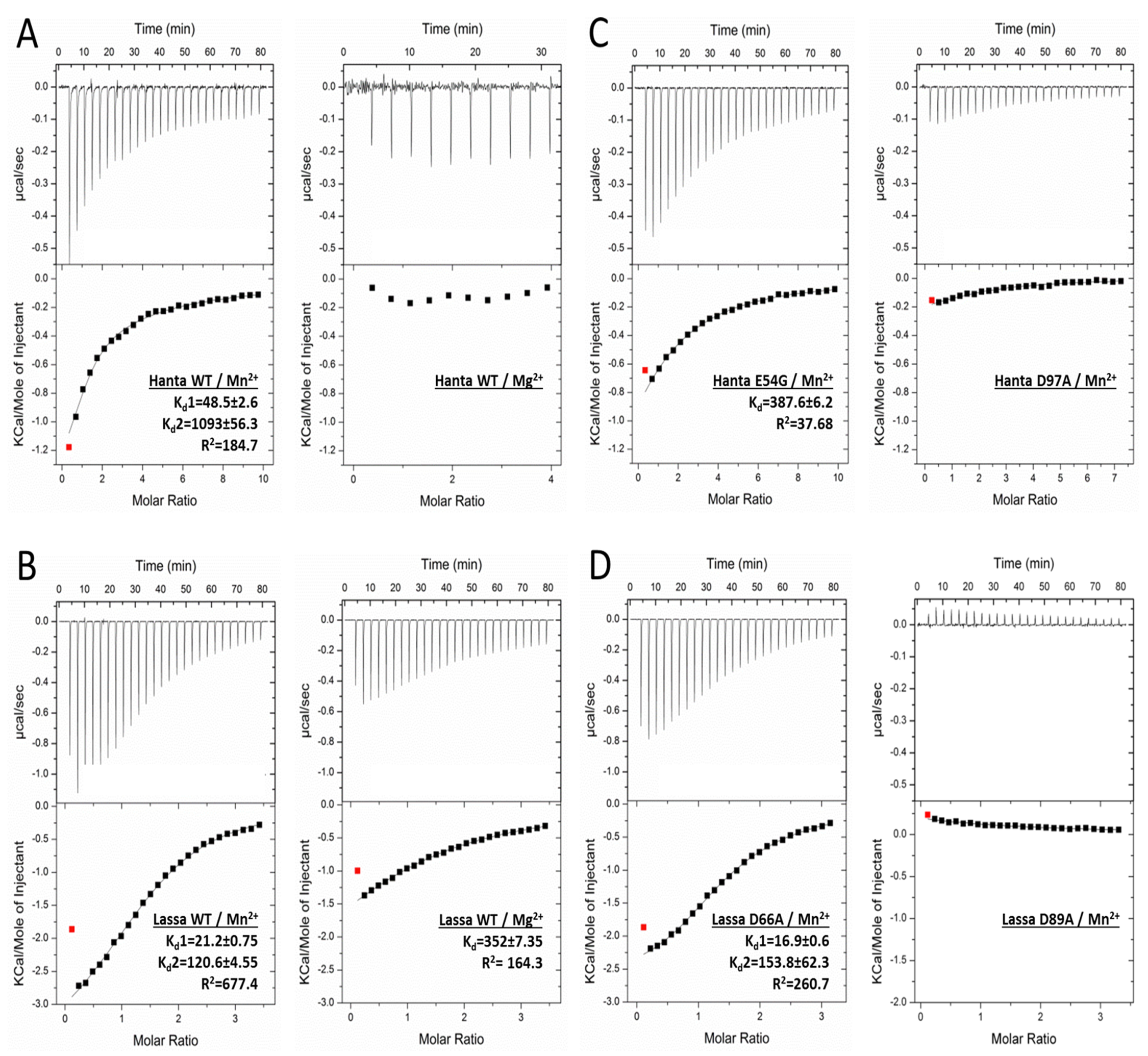

Supplement: S3 Fig — Isothermal titration calorimetry measurements.(A) manganese (left) and magnesium (right) binding to Hantaan EN wild-type (B) Manganese binding to the Hantaan EN mutants E54G (left) and D97A (right). (C) Manganese (left) and magnesium (right) binding to Lassa EN wild-type. (D) Manganese binding to the Lassa EN mutants D66A (left) and D89A (right). I all cases the upper plot shows the binding isotherm and the lower plot shoes the integrated values of each corresponding isotherm after subtracting the heat produced by the metal ion dilution. The calculated affinity values are indicated for two ion binding (Kd1 and Kd2) or one ion binding model fitting (Kd) and the fitting R2 values are indicated for each experiment. The red points were not used for the curve fitting. We used N = 1 for the one ion binding fits. (TIF) [file ppat.1005636.s003.tif]

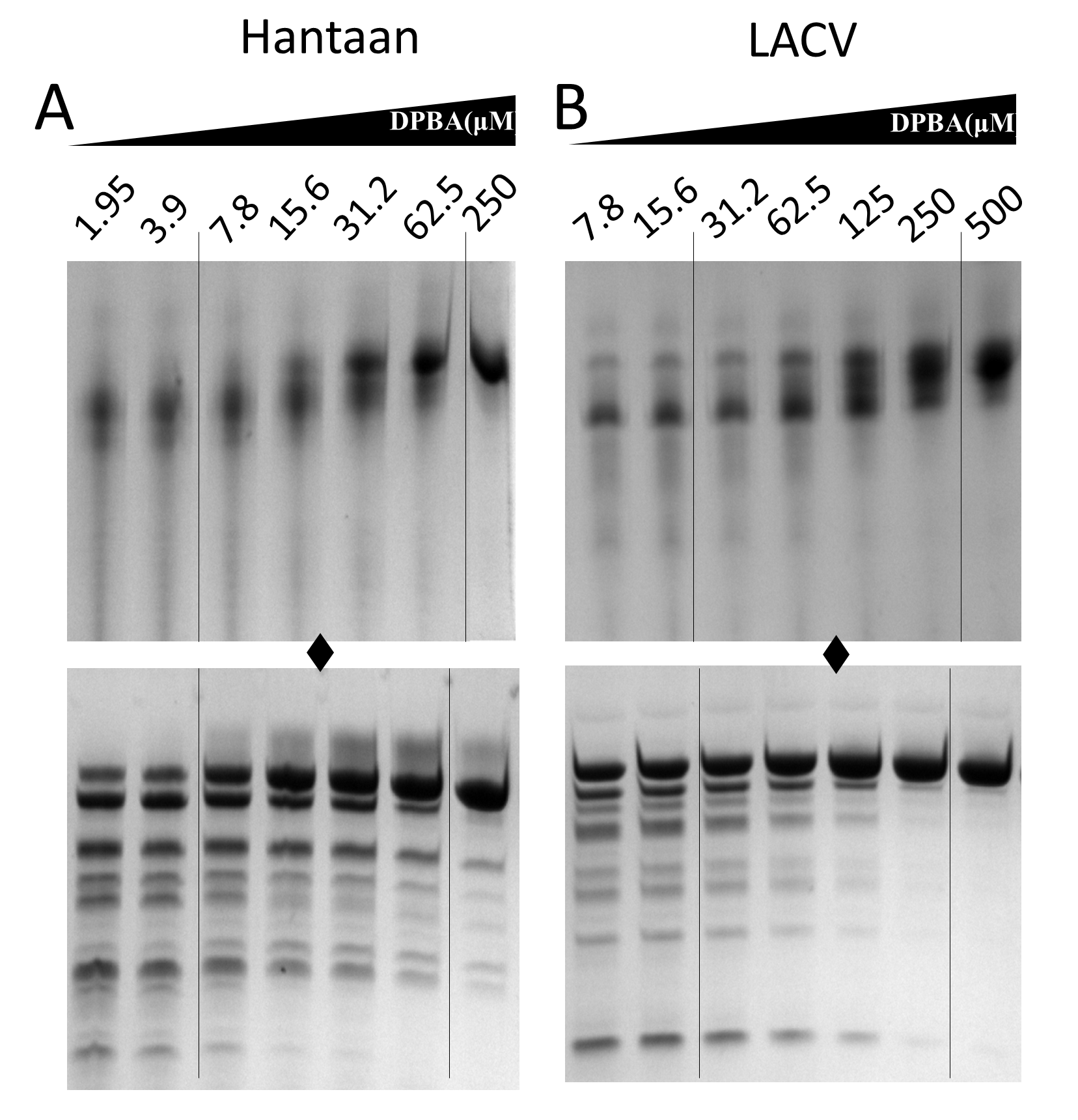

Supplement: S4 Fig — A, Denaturant Urea-acrylamide gels of nuclease reactions with 2mM MnCl2 that were carried out in the presence of increasing concentrations of DPBA for Hantaan and LACV ENs in parallel. The experiment was performed with G-rich (upper panel) and Alu SPR RNA (bottom panel). The IC50 estimated concentration is indicated with a black diamond. B, The same experiment was performed in parallel with LACV EN. (TIF) [file ppat.1005636.s004.tif]

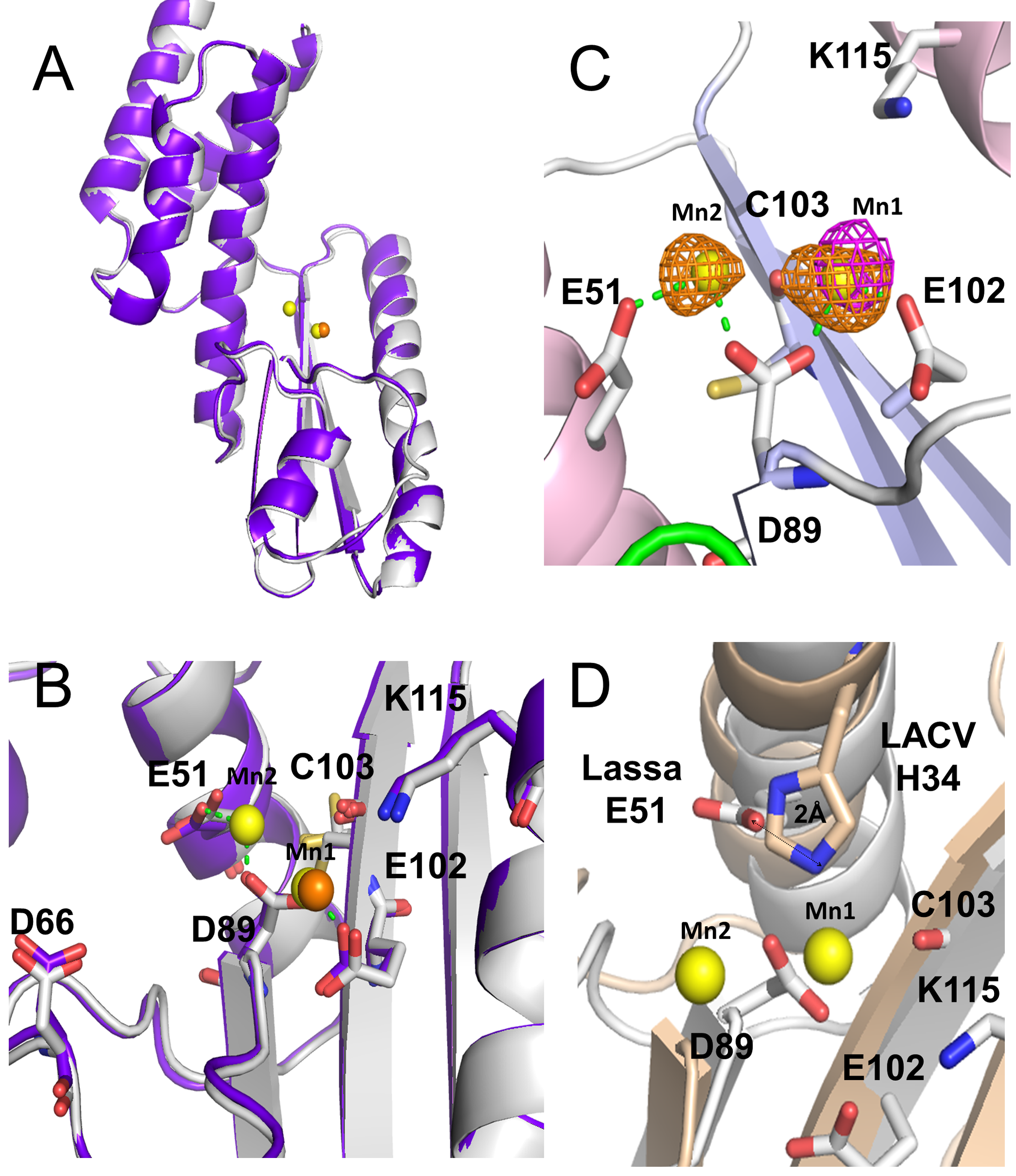

Supplement: S5 Fig — A, Superposition of Lassa X2 (grey cartoon) and X3 (purple cartoon) showing they have the same conformation. The manganese ions are represented as orange and yellow small spheres for the X2 and X3 respectively. B, Shows the same superposition as in A but focusing on the active site. The catalytic residues are represented by sticks and the Mn2+ ions by spheres as in A with the ion coordination indicated by dashed green lines. C, active site of the Lassa X3 structure shown as in Fig 3 with the anomalous difference density for X2 (purple mesh, at 50 σ) and X3 (orange mesh, at 3 σ). D, Superposition of Lassa X3 (light grey with catalytic residues as sticks) and LACV endonuclease structures (PDB: 2xi7) showing the LACV canonical position of Mn2+ ions and the catalytic histidine, highlighting the distance that E51 needs to move if it were to coordinate Mn1 as achieved by the catalytic histidine present in His+ ENs. (TIF) [file ppat.1005636.s005.tif]

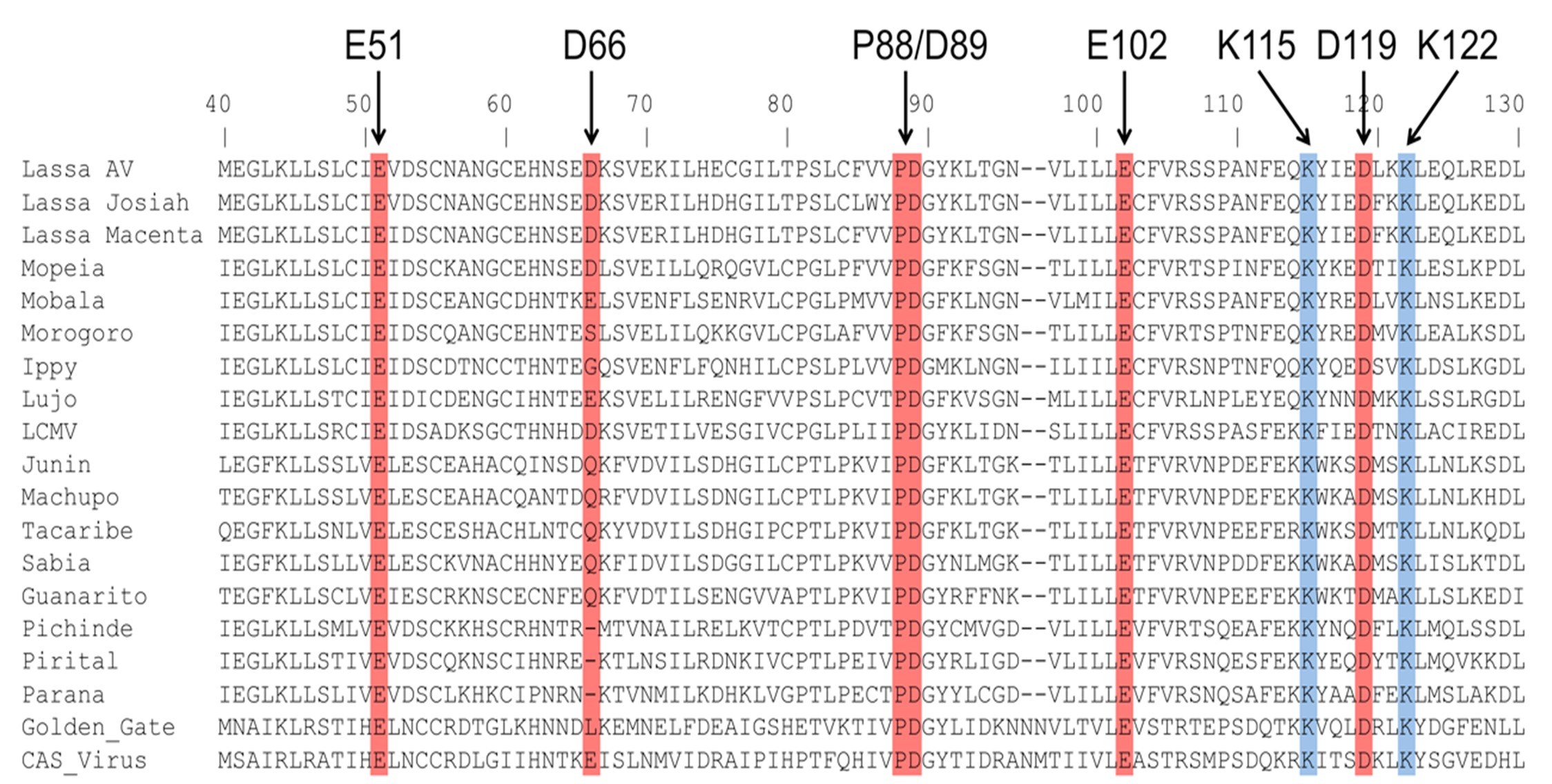

Supplement: S6 Fig — Arenavirus ENs sequence alignment indicating the catalytic residues analysed by mutagenesis in the minireplicon system. The alignment shows that D66 is not conserved as the rest of residues. (TIF) [file ppat.1005636.s006.tif]

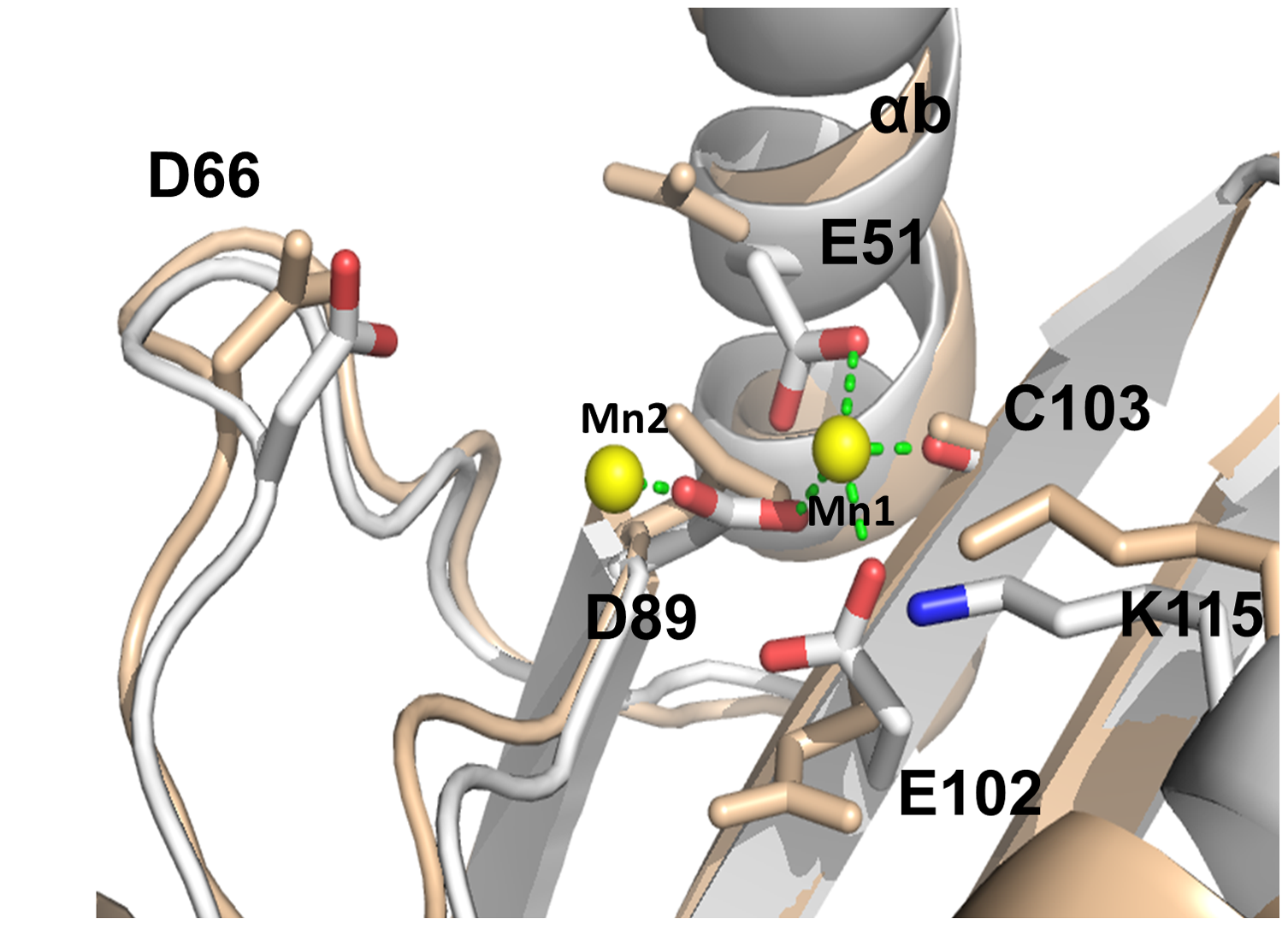

Supplement: S7 Fig — The structures of Lassa EN X1 (wheat) and the proposed model of active EN (light grey) are shown in cartoon with the active site residues shown in sticks. The two shown Mn2+ ions adopt the canonical binding like His+ endonuclease. This could be achieved by closing the active site by changing the E102 and E51 rotamers and slightly moving helix αb towards Mn1 position. (TIF) [file ppat.1005636.s007.tif]
