# Supplementary material for: Comparative Structural and Functional Analysis of Bunyavirus and Arenavirus Cap-Snatching Endonucleases
Source: PLoS Pathog. 2016 Jun 15;12(6):e1005636. doi: 10.1371/journal.ppat.1005636 (PMC4909276; doi:10.1371/journal.ppat.1005636)
Supplement: S1 Table — The molecule in the asymmetric unit used in the alignment is indicated in parenthesis for each PDB. (PDF) [file ppat.1005636.s008.pdf]

	4AVQ(A)	LassaX3(A)	Hantaan (A)
**2XI7(A)**	3.5 (122)	3.2 (135)	3.5 (127)
**4AVQ(A)**		3.7 (130)	3.6 (98)
**LassaX3(A)**			3.5 (115)
